# Interpretable AI reveals hidden structure in nuclear masses

**DOI:** 10.1038/s44488-026-00017-9

**Published:** 2026-10-21

**Authors:** Kate A. Richardson, Sokratis Trifinopoulos, Mike Williams

**Affiliations:** 1 NSF AI Institute for Artificial Intelligence and Fundamental Interactions, Cambridge, MA USA; 2 Laboratory for Nuclear Science, MIT, Cambridge, MA USA; 3 Theoretical Physics Department, CERN, Geneva, Switzerland; 4 Physik-Institut, Universität Zürich, Zürich, Switzerland

**Keywords:** Mathematics and computing, Physics

## Abstract

Predicting nuclear masses with high precision remains a central challenge in nuclear physics, with implications ranging from stellar nucleosynthesis to determining the boundaries of the nuclear landscape. Approaches using artificial intelligence (AI) have recently achieved remarkable accuracy on this task, but their usefulness is limited by their black-box nature and by the difficulty of assessing their reliability when extrapolating beyond measured nuclei. Here, we present an interpretable AI model that matches the best existing predictive precision while revealing the physical structure underlying nuclear binding energies. The model learns internal representations that form a double helix, in which links between proton and neutron embeddings correspond to the most stable nuclei within each isotopic chain. Analysis of its learned features shows that the AI autonomously recovers established macroscopic components such as the liquid-drop model and rediscovers a long-forgotten physical principle—localized Jaffe factorization—that accounts for much of its performance gains. These findings demonstrate that interpretable AI can both reproduce and extend nuclear models, uncovering latent physical symmetries directly from data.

## Introduction

For more than a century, physicists have sought to describe how the strong nuclear force binds protons and neutrons into nuclei. Despite tremendous efforts, first-principles calculations of nuclear masses—or equivalently, binding energies *E*_*b*_—are still tractable only for the lightest nuclei. Many open problems in nuclear and astro(particle) physics hinge on the lack of sufficiently precise nuclear-mass predictions. Examples include *r*-process nucleosynthesis^1^, the nuclear neutron skin and its implications on the structure of neutron stars^2–4^, the exploration of the boundaries of the nuclear landscape^5^, and exotic phenomena such as halo nuclei^6^ and shape coexistence^7,8^. Experimental measurements are restricted to (quasi)stable nuclei^9^, leaving large regions of the nuclear landscape inaccessible except through theoretical inference.

Microscopic-macroscopic approaches, such as the most recent Weizsäcker-Skyrme (WS4) model^10^, currently provide the most precise theoretical predictions. In these frameworks, extended liquid-drop (LD) macroscopic terms are supplemented by microscopic corrections calculated within a mean-field approximation. Despite their success, such models remain limited by simplifying assumptions in the microscopic part that are required to keep the many-body problem computationally tractable. As a result, even their most refined versions fall short of the precision needed to fully resolve key astrophysical and nuclear-structure questions^11–13^.

In recent years, artificial intelligence (AI) has emerged as a promising alternative^14–31^. Machine-learning models have been developed that either augment existing physics-based frameworks by learning residual corrections or attempt fully data-driven predictions with no explicit physics input. Some of these models now achieve precision surpassing the best physics-based ones. Yet their reliability remains uncertain: predictions are most valuable where no measurements exist, requiring extrapolation beyond the region covered by the training data. Since most neural networks (NNs) are effectively black boxes, it is unclear whether their predictions can be trusted far from the stability regime. This raises a central question: *What is AI actually learning about nuclei?*

Here we present an AI model—a simplified variant of our previous framework^24,25^—that achieves state-of-the-art precision in predicting nuclear masses while remaining fully interpretable. The model learns internal representations of proton and neutron numbers, whose dominant dimensions form a double helix, revealing how data-driven learning captures nuclear stability. Decomposition of the learned features shows that the AI predictions can be hierarchically factorized, with the leading terms corresponding to known macroscopic models such as the LD formula^32,33^. Remarkably, the model’s improvement over symbolic approaches originates almost entirely from a rediscovery of *localized Jaffe factorization*^34^, a long-overlooked principle that explains much of the residual structure in nuclear masses. The result is a transparent, data-driven framework that unifies empirical accuracy with physical insight, demonstrating how interpretable AI can expose the underlying symmetries and hierarchies of nuclear structure.

## Results and discussion

### Background: physics models of nuclear masses

Before discussing the AI model, we briefly review the history and status of physics models that predict *E*_*b*_. The LD model, proposed almost a century ago, treats the nucleus as a highly dense incompressible fluid consisting of *Z* protons and *N* neutrons, thus

1 $$\begin{array}{lll}{E}_{b}^{{{{\rm{LD}}}}}\approx {\alpha }_{v}A-{\alpha }_{s}{A}^{2/3}-{\alpha }_{c}\frac{Z(Z-1)}{{A}^{1/3}}\\ \hspace{0.5em}-{\alpha }_{a}\frac{{\left(N-Z\right)}^{2}}{A}+{\alpha }_{p}\frac{\delta (Z,N)}{{A}^{1/2}},\end{array}$$

where *A* = *Z* + *N* is the total number of nucleons (protons and neutrons) in the nucleus. The first three terms account for: (volume) the nuclear energy being proportional to the volume of the incompressible fluid due to the short-range nature of the strong force, (surface) a correction to the previous term due to nucleons near the surface having fewer nearby nucleons to interact with, and (Coulomb) the electromagnetic potential energy of each pair of protons. The last two terms, asymmetry and pairing, both arise due to the Pauli Exclusion Principle. The pairing term always favors *Z* and *N* being even over odd, though the precise form of *δ*(*Z*, *N*) varies. Despite its simplicity, after fitting the *α*_*i*_ coefficients to data, the LD predictions achieve a typical precision of ${{{\mathcal{O}}}}( \% )$ for moderate nuclei and ${{{\mathcal{O}}}}(0.1 \% )$ for large nuclei^35^, which is, e.g., about a factor of 30 from the precision required for *r*-process calculations^13^. Supplementary Fig. S1 in our Supplemental Material compares ${E}_{b}^{{{{\rm{LD}}}}}$ to data.

Over the decades, many additional terms, along with modifications to the terms in Eq. (1), have been proposed, though there is no universally accepted improved symbolic formula. This is in part due to the near degeneracy of many proposed functions of *Z* and *N* over the small region of the (*Z*, *N*) plane where measurements exist. Roughly, the precision can be improved by a factor of two by increasing the number of parameters by a factor of three (relative to the basic LD model)^35^, which is still an order of magnitude away from the target precision.

While the LD model captures global trends, more sophisticated microscopic-macroscopic frameworks have been developed to incorporate shell effects and other quantum corrections^10,36–40^. The microscopic part refers to calculations of single-nucleon energy levels using the approximation that all nucleons experience a mean-field potential due to their interactions with all other nucleons in the nucleus^41–49^. The potential used is phenomenological in nature with a number of parameters determined by fitting the available nuclear data. The macroscopic part refers to how these single-nucleon energy levels collectively vary across the nuclear (*Z*, *N*) plane, e.g., following an equation similar to Eq. (1). Therefore, in this family of models, the macroscopic part is a symbolic expression, while the microscopic part is the output of a simplified many-body quantum-mechanical calculation. Currently, the WS4 model provides the best precision with a root-mean-square (RMS) error of about 0.28 MeV^10^ (all RMS values quoted here are calculated on the same set of nuclei as described in the Supplemental Material). The macroscopic expression, provided in Supplementary Eq. (S8), is a slightly modified version of Eq. (1) that has 9 parameters. The potential used in the microscopic calculations is an *axially deformed Woods-Saxon* potential, see ref. ^10^ for details. Supplementary Fig. S1 compares the WS4 predictions to experimental data. The limitations of even the best physics-based model motivate exploring whether data-driven methods can identify similar (or even improved) physical structure directly from measurements.

### AI model overview and physical analogy

In ref. ^24^, we presented an AI model that predicts various nuclear observables, including *E*_*b*_, separation energies, and charge radii. The model was trained using a multi-task learning approach, where a common internal representation was learned to predict the full set of nuclear observables by leveraging their joint information. No inductive bias or physics inputs were used, thus these AI predictions are entirely empirical, i.e. they are based only on patterns learned directly from the available nuclear data. Despite this lack of physics awareness, our AI model was able to achieve an exceptional precision of 0.13 MeV, a mean relative precision of ${{{\mathcal{O}}}}(1{0}^{-4})$. The primary focus of ref. ^24^, and a follow-up work^25^, was AI-centric, specifically how simultaneously learning to perform multiple tasks can improve performance, and that high-dimensional NNs can learn interpretable low-dimensional representations; whereas, our focus here is physics-centric, namely is what the AI model learned useful for science?

To understand what physical principles the AI model has learned, we analyze the internal representations formed during training. We study a simplified version of the model in refs. ^24,25^ that is only trained to predict *E*_*b*_ and only has access to *E*_*b*_ measurements. There are some aspects about the model architecture and learning dynamics that are important in understanding what the model has learned. We present these here in a physics-centric way, with the full details in Supplemental Material. The only inputs for each nucleus are *Z* and *N*, which we promote to 1024-dimensional vectors that are fed into a NN, *F*_nn_, with learnable parameters $\vec{\theta }$:

2 $$Z\to \vec{Z},N\to \vec{N}\Rightarrow {E}_{b}={F}_{{{{\rm{nn}}}}}(\vec{Z},\vec{N},\vec{\theta }).$$

This architecture was chosen to retain the potential interpretability of learned proton and neutron embeddings, while giving the model enough capacity to make high-precision predictions. The vectors $\vec{Z},\vec{N}$ are initialized randomly and learn to embed properties about each number of protons and neutrons through training. This includes relative properties which we will show can be stored, e.g., in the angles between components of the vectors. More explicitly, for each unique integer *Z* or *N* there is a corresponding 1024-dimensional vector $\vec{Z}$ or $\vec{N}$ whose components are independently variable during training (regardless of how many nuclei with a given *Z* or *N* are used during training, there is always only one $\vec{Z}$ and $\vec{N}$ embedding vector for each unique *Z* and *N* value). The objective during training is to minimize the following loss function:

3 $${{{\mathcal{L}}}}={\sum}_{i}{\left({E}_{b,i}^{{{{\rm{ex}}}}}-{E}_{b,i}^{{{{\rm{ai}}}}}\right)}^{2}+\lambda \,\left[{\sum}_{j}{Z}_{j}^{2}\,+\,{\sum}_{k}{N}_{k}^{2}\,+\,{\sum}_{\ell }{\theta }_{\ell }^{2}\right]\,,$$

where ${E}_{b,i}^{{{{\rm{ex}}}}}$ and ${E}_{b,i}^{{{{\rm{ai}}}}}$ are the experimental and AI-predicted *E*_*b*_ values for each nucleus in the training data, and *λ* is a hyperparameter that controls the balance between prediction quality and model complexity. Here, *j* runs over all components of all learned $\vec{Z}$ embeddings and *k* runs over all components of all learned $\vec{N}$ embeddings; these sums are not over the integer proton and neutron numbers themselves.

### Emergent double-helix representation

**Fig. 1:**
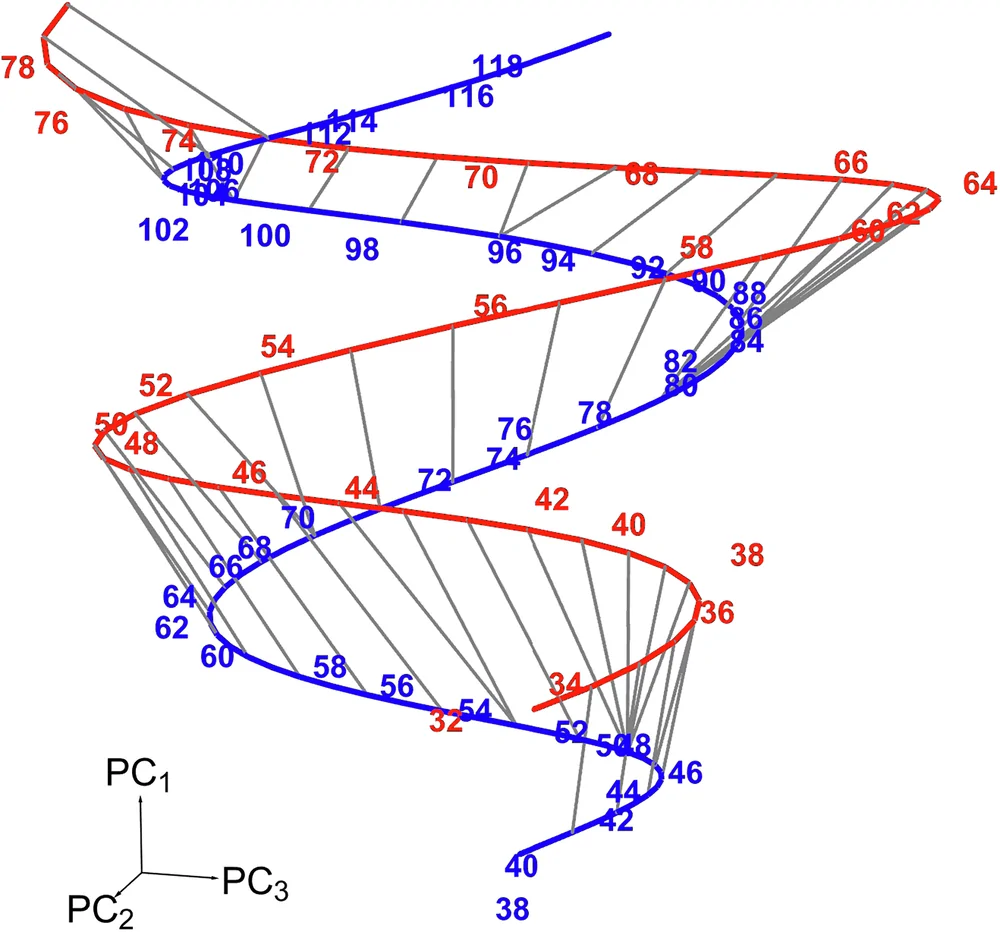
Learned proton and neutron embeddings form a double helix. The three most important *principal components* (PCs) of the internal representations learned by our AI model for (red) proton number *Z* and (blue) neutron number *N*. Links connect the values of *Z* and *N* found in the most stable nucleus of each isotopic chain. Shown is the region of (*Z*, *N*) where the nuclear binding energy, *E*_*b*_, is well described by the volume and asymmetry terms in Eq. (1); only even values are labeled to avoid clutter. The curves show helical fits to the *Z* and *N* representations, revealing that the model encodes proton-neutron coupling in a double-helix geometry analogous to DNA.

We first analyze the learned representations of *Z* and *N* to identify what physical information the model has encoded here. From a physics perspective, this AI model is a system with degrees of freedom {*Z*_*j*_, *N*_*k*_, *θ*_*ℓ*_} initialized in a random configuration, where the training procedure implements a dissipative process to find the ground state, defined as the one with the minimum ${{{\mathcal{L}}}}$. The regularization term in Eq. (3), the *λ*[…] term, encourages efficient storage of information in the components of $\vec{Z},\vec{N}$. In essence, it attaches to each AI model degree of freedom an oscillator with zero natural length and spring constant 2*λ*. We can rank directions in $\vec{Z},\vec{N}$ space by how much projections to those axes contribute to the *E*_*b*_ predictions using a technique known as principal component analysis (PCA). Figure 1 shows the three most important principal components (PCs) of $\vec{Z},\vec{N}$, denoted ${{{{\mathcal{Z}}}}}_{1,2,3},{{{{\mathcal{N}}}}}_{1,2,3}$, where a clear double-helix structure has formed. These emergent structures, whose formation is found to be robust provided regularization is applied (the architecture choices and robustness checks are summarized in the Supplemental Material. Within this regularized learned-embedding family, the double-helix geometry is robust across the hyperparameter variations explored in our code and earlier studies^24,25^; removing regularization can instead yield an ordered representation with comparable performance but without the same helical geometry^25^) suggest that the model organizes nuclear information in a low-dimensional, physically meaningful space—a prerequisite for interpretable decomposition.

There is a resemblance between the structure formed in ${{{\mathcal{Z}}}}$-${{{\mathcal{N}}}}$ space and that of DNA, which forms a compact double helix to minimize energy. Within the AI architecture employed here, the double helix is a compact geometrical structure that minimizes the loss (the analogy to DNA is illustrative but not fundamental to nuclear structure; it is tied to the choice of using learned embeddings and regularization. That said, within this family of AI models the emergence of a double helix is robust (see Supplemental Material). The nucleotide bases in DNA are hydrophobic, and pushed inwards by the surrounding water in the cell. In our AI model, the regularization term provides the force that attracts the elements inwards. In DNA, Van der Waals forces oppose the hydrophobic pressure, whereas in our AI model this effect is driven by the need to fit the data well to minimize the error term in ${{{\mathcal{L}}}}$, i.e. to provide good predictions of *E*_*b*_. The volume term is dominant in Eq. (1), hence *E*_*b*_ ≈ *α*_*v*_*A* and our AI model must be able to build *A* from $\vec{{{{\mathcal{Z}}}}}$ and $\vec{{{{\mathcal{N}}}}}$. This drives the most important PCs to be (see Fig. 1 vertical)

4 $${{{{\mathcal{Z}}}}}_{1}\approx \beta Z,{{{{\mathcal{N}}}}}_{1}\approx \beta N\to A\approx ({{{{\mathcal{N}}}}}_{1}+{{{{\mathcal{Z}}}}}_{1})/\beta ,$$

where *β* is a scale factor and the addition operation is trivial for the downstream NN to implement. To produce the volume term, the NN needs to store the factor *α*_*v*_/*β* in its $\vec{\theta }$ parameters. Note that while the regularization term on {*Z*_*j*_, *N*_*k*_} wants to drive *β* → 0, this results in a contribution from the {*θ*_*ℓ*_} regularization of *α*_*v*_/*β* → *∞*. As we show in detail in the Supplemental Material, there is a non-zero value of the scale of ${{{{\mathcal{Z}}}}}_{1},{{{{\mathcal{N}}}}}_{1}$, that minimizes ${{{\mathcal{L}}}}$ for a given set up, thus compressive regularization pressure is, as expected, counterbalanced by the goodness-of-fit term in ${{{\mathcal{L}}}}$.

The same reasoning that prevents the first PC from collapsing under the regularization pressure also applies to the second and third PC dimensions shown in Fig. 1. However, this does not explain the origins of the helical structure. Naively, there are no cyclic or oscillatory terms in Eq. (1), making it surprising that the second and third most important PCs form an oscillatory structure. The explanation lies in the second most important term in Eq. (1), the asymmetry term, which contains a factor of (*Z*−*N*)^2^. This term is invariant under *Z* → *Z* + *γ*, *N* → *N* + *γ*, i.e. translations along the ${\hat{{{{\mathcal{Z}}}}}}_{1}$ and ${\hat{{{{\mathcal{N}}}}}}_{1}$ axes (which are nearly aligned) in Fig. 1. When computing (*Z*−*N*)^2^, only the relative difference of *Z* and *N* matters; therefore, the same solution can be repeated all along the first PC axis. We show in the Supplemental Material precisely how the double helix is used to obtain (*Z*−*N*)^2^ efficiently (see also Supplementary Figs. S3, S4). Therefore, the helical geometry is a compact way for this architecture to represent the dominant volume coordinate together with the relative proton-neutron displacement entering the asymmetry term. Other architectures—or even this one without regularization—can represent these same aspects of nuclear structure using alternative geometries. Electromagnetic interactions of protons break the symmetry between *Z* and *N*, which is why the double helix in Fig. 1 is not symmetric. A symmetric double helix is obtained when the experimental data is replaced with simulated isospin-symmetric data (see Supplemental Material).

### Hierarchical decomposition of binding energy

**Fig. 2:**
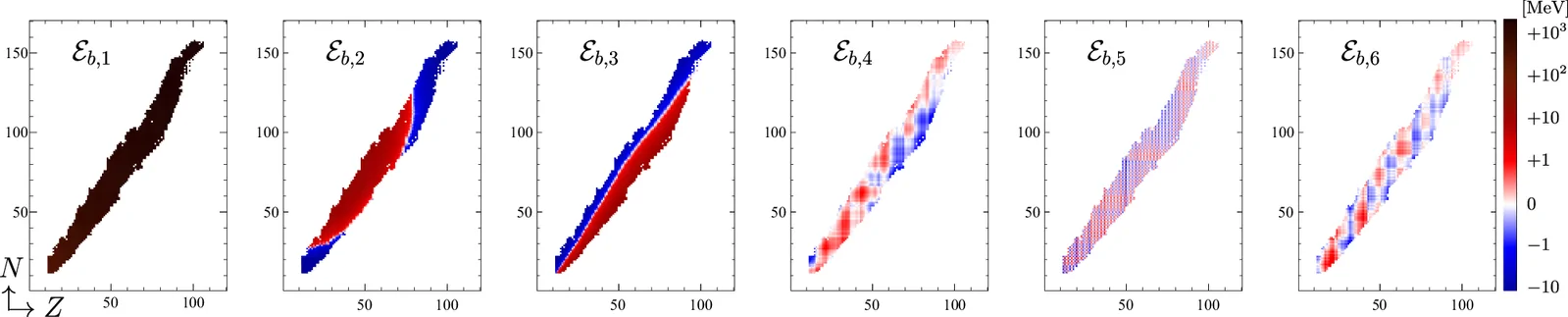
Principal components of the AI prediction reveal a macro-to-micro hierarchy. The six most important principal components (PCs) of the AI nuclear binding energy, *E*_*b*_, predictions, shown as contributions ${{{{\mathcal{E}}}}}_{b,i}$ over the nuclear (*Z*, *N*) plane, where *Z* and *N* are the proton and neutron number. Color indicates the contribution in MeV. The scale is linear on [−1, 1] and logarithmic otherwise. The first three PCs are approximately smooth functions and largely correspond to macroscopic terms, whereas the lesser PCs are discrete and approximately factorize as *F*_*Z*_(*Z*) + *F*_*N*_(*N*), where *F*_*Z*_ and *F*_*N*_ are functions of only proton number *Z* and neutron number *N*, respectively, illustrating the transition from macroscopic to microscopic structure.

An advantage of our AI approach—embedding information about proton and neutron number into learnable vectors—is that structures formed in the PC space correlate strongly with predictive power of the model^25^. The PCs are defined such that the most-important components of $\vec{{{{\mathcal{Z}}}}}$ and $\vec{{{{\mathcal{N}}}}}$ by construction affect the most nuclei; structure in these components maps to the macroscopic terms in physics models. The fact that there are preferred directions entering the NN, corresponding to the most important $\vec{{{{\mathcal{Z}}}}}$ and $\vec{{{{\mathcal{N}}}}}$ directions, results in a hierarchy forming in the penultimate NN layer, where the terms that are summed to make the *E*_*b*_ predictions are built (we focus on the embedding space and the penultimate layer because they have direct interpretability roles: the former shows how *Z* and *N* are represented, while the latter is closest to the additive decomposition of the final prediction. Other hidden layers may also contain structure, but we do not study these here). Figure 2 shows that the most-important PCs in this space, denoted ${{{{\mathcal{E}}}}}_{b,i}$, are approximately smooth functions; in fact, they are well described by known symbolic nuclear models (see Supplemental Material). The first three PCs, therefore, largely describe macroscopic terms (because the PCs are orthogonal while LD terms are not, this correspondence should be understood as an approximate hierarchy of dominant physical trends rather than a one-to-one decomposition of the LD formula). The lesser PCs correspond to microscopic terms, providing corrections for various energy levels.

The form of the lesser PCs in Fig. 2 is worthy of discussion. A decomposition of these leading PCs into macroscopic and microscopic components, together with fits of the symbolic model, is shown in Supplementary Fig. S7. Recall that the microscopic terms in physics models are calculated using a mean-field many-body quantum-mechanical approach. However, the AI model has instead, to a good approximation, learned a much simpler estimate for microscopic terms:

5 $${E}_{b}^{{{{\rm{mic}}}}}\approx {F}_{Z}(Z)+{F}_{N}(N)\,,$$

where *F*_*Z*,*N*_ are discrete functions of only *Z* or *N*, and importantly, not both; *i.e*. the microscopic terms approximately factorize, making them easy to learn. For example, our Supplemental Material shows that fitting a higher-order PC with a characteristic 100 keV scale to *F*_*Z*_(*Z*) + *F*_*N*_(*N*) leaves only a 4 keV residual RMS (see Supplementary Fig. S6).

Remarkably, this same factorization observation was made in 1969^34^, though it seems to have been forgotten until rediscovered by our AI model. Reference ^34^ was based on the Garvey-Kelson (GK) relations, patterns found in nuclear data where combinations of *E*_*b*_ from a set of nuclei approximately cancel almost anywhere in the nuclear plane (examples in Supplemental Material). The GK relations arise because single-nucleon energy levels do not typically vary much in a small region of the nuclear plane. Any contribution to *E*_*b*_ that varies slowly over the nuclear plane, e.g., macroscopic terms like Eq. (1), approximately cancels in the GK relations. However, microscopic terms that exactly satisfy the full set of GK relations studied in ref. ^34^ are severely restricted, they must factorize as in Eq. (5). We henceforth refer to this as *Jaffe factorization*. Examples of the Garvey-Kelson relations in the (*Z*, *N*) plane are shown in Supplementary Fig. S5.

Dissecting the leading PCs ${{{{\mathcal{E}}}}}_{b,i}$ to determine which symbolic terms to use in a macroscopic component, ${E}_{b}^{{{{\rm{mac}}}}}$, we find that the same modified version of Eq. (1) used by the WS4 model, except replacing the Wigner term by ${E}_{b}^{W}={\alpha }_{W}| Z-N| /A$, works well. This Wigner term has been considered in refs. ^35,40^, but is not included in many other popular macroscopic formulas. Next, we consider a simple model using the macroscopic terms obtained from our study of the leading AI model PCs, along with Jaffe factorized microscopic terms as in Eq. (5). We find that this simple model works remarkably well, except near the double-magic nuclei such as ^208^_82_Pb. The fact that Jaffe factorization breaks near these double-magic nuclei is not surprising, so we adopt dedicated terms for these regions (see Supplemental Material). The local smoothness assumption underlying the GK relations is weakest near large shell gaps, where single-particle level densities and shell-correction contributions can change rapidly when adding or removing a few nucleons. The dedicated double-magic terms should, therefore, be viewed as phenomenological markers of the small populated regions where local factorization breaks. After including these double-magic terms, our simple model achieves an RMS accuracy of 0.37 MeV; see Supplementary Fig. S8. Therefore, most of the improvement of the AI model over the macroscopic terms is captured (note that terms accounting for nuclear shell structure and magic numbers factorize, hence are implicitly included via Eq. (5)). The AI model thus rediscovered a hierarchy long recognized in symbolic physics models—from macroscopic liquid-drop terms to microscopic shell corrections—culminating in a factorized form first identified by Jaffe. This hierarchy of learned components provides the foundation for the local corrections discussed next.

### Local Jaffe factorization and predictive performance

**Fig. 3:**
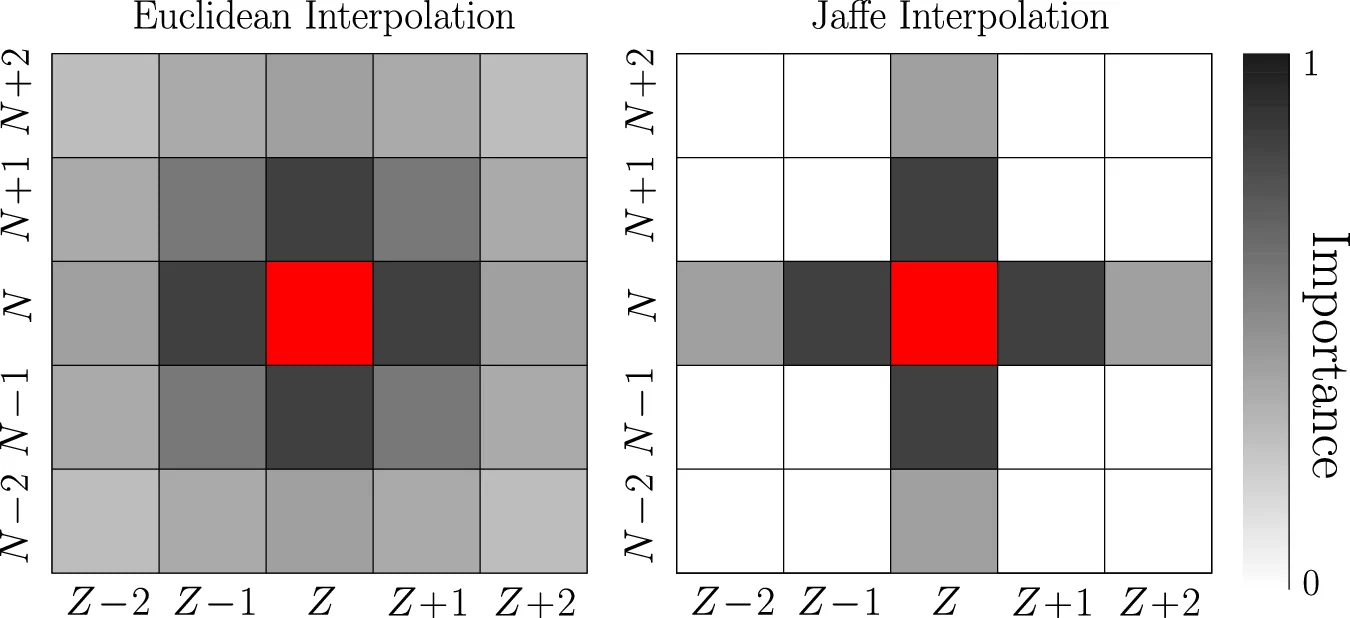
Local Jaffe interpolation selects physically informative neighbors. Relative importance (shading) of neighboring nuclei using (left) Euclidean (purely distance-based) and (right) local Jaffe interpolation (target nucleus at center, colored red; *Z* and *N* denote proton and neutron number, respectively). The Jaffe scheme emphasizes nuclei with identical proton or neutron numbers, showing that physically informed neighborhood structure outperforms simple geometric distance.

Jaffe factorization in Eq. (5) is overly restrictive since the GK relations only hold *locally*, i.e. in small regions of the nuclear plane, whereas Eq. (5) applies global corrections for each *Z* and *N*. We can relax Jaffe factorization and apply it locally using the observed *E*_*b*_ values of neighboring nuclei. Indeed, many models are based on learning local corrections to WS4 based on the distances to observed neighboring nuclei (see Fig. 3 (left)), achieving excellent precision, e.g. an RMS of 0.18 MeV in ref. ^10^. However, local Jaffe factorization implies that distance in the nuclear plane is not the most important consideration. Instead, neighbors with either the same *Z* or *N* are the most useful. We find that corrections obtained from these neighbors, arranged as a +, not only outperform the remaining neighbors, arranged in an ×—but the ×corrections provide no improvement ((Jaffe factorization implies that the error incurred by using  × neighbors is twice that of the +neighbors (see Supplemental Material). Even using next-to-nearest-neighbor + nuclei dramatically outperforms nearest-neighbor × nuclei.

Local Jaffe corrections using the +nearest and next-to-nearest neighbors as in Fig. 3 (right) reduces the RMS of our simple model, which recall has no physics microscopic terms, to 0.18 MeV. This is substantially better than WS4, the best physics model, and even consistent with ref. ^10^. Instead, applying these corrections to WS4 reduces its RMS to 0.16 MeV, which beats all but the most advanced AI models (such as ref. ^24^) without using any AI or complicated interpolation.

**Fig. 4:**
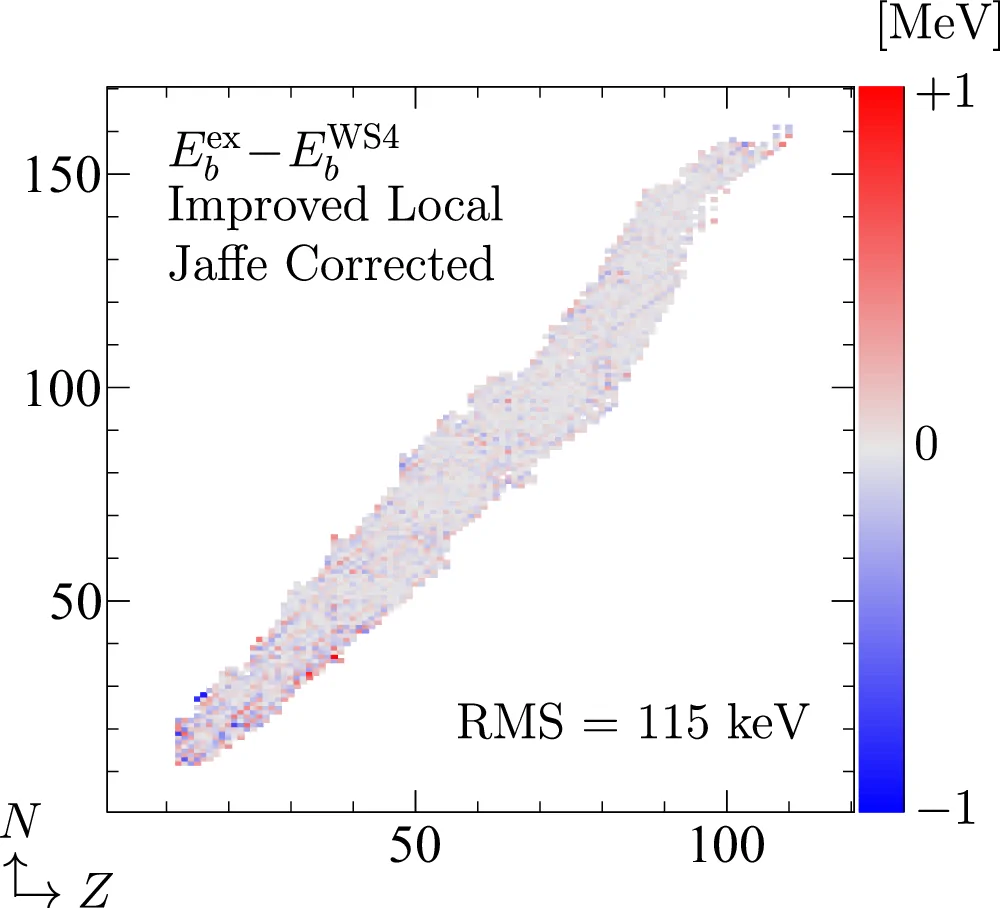
Improved local Jaffe corrections reduce WS4 residuals to the 100 keV scale. Residuals between the experimental measurements and the WS4 model including the improved local Jaffe corrections, ${E}_{b}^{{{{\rm{ex}}}}}-{E}_{b}^{{{{\rm{WS4}}}}}$. The overall RMS of 115 keV demonstrates that interpretable local corrections achieve state-of-the-art precision comparable to advanced AI models.

Local Jaffe corrections can be improved by also correcting the neutronic (protonic) component for isotopic (isotonic) neighbors, which are used to obtain the protonic (neutronic) corrections in Fig. 3 (right). This is done by considering all available nearest and next-to-nearest neighbors of nucleus (*Z*, *N*) in a linear regression that determines local Jaffe corrections for each *Z* ∈ [*Z* − 2, *Z* + 2] and *N* ∈ [*N* − 2, *N* + 2] (local Jaffe corrections work well even when most neighboring nuclei are unobserved, which is a major advantage over using the GK relations directly; see Supplementary Table S1. In particular, within the measured domain, the local Jaffe method remains accurate along the neutron-rich edge of the occupied (*Z*, *N*) plane, especially for *Z*, *N* ≳ 50, with several larger residuals at lower masses mostly concentrated near magic and double-magic nuclei, where enhanced shell effects are expected, see Supplemental Material). Applying these corrections to WS4 produces an RMS below 0.12 MeV (residuals shown in Fig. 4) (for broader context, a comparison of contemporary AI nuclear-mass benchmarks is collected in Fig. 5 of ref. ^50^). These are state-of-the-art results for *E*_*b*_ predictions—and they are based on explainable corrections to a physics model, not a black-box AI approach. Therefore, our main result is that AI has rediscovered a long forgotten nuclear property we refer to as Jaffe factorization, and when this is applied locally to physics-model calculations it provides state-of-the-art, interpretable predictions for nuclear masses.

The GK relations exemplify the type of empirical patterns that an AI model is naturally predisposed to learn. Within our framework, the AI effectively rediscovers these relations and encodes them through localized Jaffe factorization, translating empirical patterns into physically interpretable structure. More precisely, the role of AI in this chain was that the structure of the lesser *E*_*b*_ PCs of the AI prediction exposed an approximate local factorization down to the scale of the precision of the model itself. Imposing this factorized structure directly led to the local Jaffe correction scheme. This mechanistic connection explains why our model’s local corrections outperform conventional interpolation schemes and why its improvements over symbolic models remain grounded in well-understood nuclear structure physics.

## Conclusions

These results demonstrate that interpretable AI can achieve state-of-the-art precision in predicting nuclear binding energies while offering genuine physical insight into the learned representations. Our model reveals that the dominant internal dimensions form a double helix, encoding a deep correspondence between the learned embeddings and the proton-neutron coupling in (quasi)stable nuclei.

Remarkably, the improvement of our model over existing symbolic approaches can be traced to a rediscovery of localized Jaffe factorization, a long-overlooked property originally derived from the Garvey-Kelson relations. This finding shows that data-driven models can independently recover latent physical laws and hierarchies, bridging the gap between symbolic nuclear theory and empirically derived rules. Importantly, these results provide, to our knowledge, the first evidence in a theoretical-physics setting that AI interpretability does not come at the expense of accuracy; with an appropriate architecture, both can be realized simultaneously.

Local Jaffe factorization also reframes an important question for future work: Does this same nuclear-structure property hold in the *r*-process regime, where extrapolation far from stability is essential? Thus, our work transforms the question from whether an AI model trained on (quasi)stable nuclei can predict *r*-process masses to the physical question of how far local Jaffe factorization remains valid beyond the nearest experimental anchors toward the relevant neutron-rich nuclei. Answering this is not a purely empirical problem of the kind studied here; it requires nuclear-theory input on the structure of these nuclei and their associated uncertainties. A quantitative assessment in the *r*-process regime is, therefore, a substantial problem that warrants a dedicated investigation.

More broadly, this work illustrates how human-AI collaboration can uncover hidden structures and accelerate discovery in the physical sciences. By combining data-driven modeling with interpretable representations, AI can move beyond black-box predictions to reveal the governing relations underlying complex systems. The same framework that we used here—embedding physically meaningful quantities into learnable vector spaces—could be applied to a wide range of scientific problems. By revealing hidden structure in nuclear mass models, interpretable AI provides a transparent foundation for both prediction and understanding, transforming data-driven computation into a tool for scientific reasoning.

## Methods

We use binding-energy data from the AME2020 Atomic Mass Evaluation^9^, restricting the evaluation to nuclei with *Z*, *N* ≥ 12 and excluding nuclei with mass uncertainties above 100 keV or masses determined from mass-surface analysis. This gives 2325 nuclei with 12 ≤ *Z* ≤ 118 and 12 ≤ *N* ≤ 178. Quoted root-mean-square (RMS) errors are unbiased: the binding energy of each evaluated nucleus is excluded from the data used to determine the parameters entering its prediction. Further details on the dataset and evaluation protocol are given in the Supplemental Material.

The AI model maps each allowed proton number *Z* and neutron number *N* to independently learned 1024-dimensional embedding vectors. These embeddings are concatenated and passed through a fully connected layer, two residual blocks, and a linear readout to predict the binding energy *E*_*b*_ (Supplementary Fig. S2). The model is trained with an *L*_2_-regularized squared-error loss using AdamW; the regularization acts on the embedding components and neural-network weights. Details of the architecture, hyperparameters, and robustness checks are given in the Supplemental Material.

To interpret the trained model, we apply principal component analysis (PCA) to both the learned *Z*, *N* embeddings and the penultimate-layer contributions to the binding-energy prediction. The leading embedding components are used to identify the double-helix representation, while the penultimate-layer components are decomposed into smooth macroscopic terms and discrete terms that approximately factorize into the form *F*_*Z*_(*Z*) + *F*_*N*_(*N*).

Local Jaffe corrections are evaluated without using the target nucleus. Basic corrections use measured nearest and next-to-nearest isotopic and isotonic neighbors, while the improved correction fits local protonic and neutronic shifts by linear regression over the available neighboring nuclei. The complete definitions of these procedures and comparisons to Garvey-Kelson corrections are provided in the Supplemental Material.

## Supplementary information

Supplementary information

Supplementary Data 1

## Data Availability

No datasets were generated or analysed during the current study.

## Code availability

The exact code version used for model training, analysis, and figure generation is archived at Zenodo^51^ and released under the MIT License. The development repository is available at https://github.com/strifinopoulos/Nuclear_DNA.
