# Supplementary material for: Interpretable AI reveals hidden structure in nuclear masses
Source: Commun AI Comput. 2026 Oct 21;1(1):20. doi: 10.1038/s44488-026-00017-9 (PMC13686942; doi:10.1038/s44488-026-00017-9)
Supplement: Supplementary file 1 [file 44488_2026_17_MOESM1_ESM.pdf]

Interpretable AI Reveals Hidden Structure in Nuclear Masses

Supplemental Material

Kate A. Richardson, Sokratis Trifinopoulos, and Mike Williams

DATA SET & DEFINITION OF PERFORMANCE METRIC

We use data from the latest Atomic Mass Evaluation (AME2020) data set [9]. When quoting RMS errors, we focus on nuclei with $Z, N \geq 12$, since small nuclei are each distinct enough that it is difficult to infer their properties directly from other nuclei, *i.e.* without physics inputs. We also remove from the data sample poorly measured nuclei—those with a precision worse than 100 keV or those determined with mass surface analysis— since comparing to measured values with uncertainties larger than our target precision introduces unnecessary noise. This leaves 2325 nuclei with $12 \leq Z \leq 118$ and $12 \leq N \leq 178$.

The RMS values we quote are unbiased, each nucleus is masked (not considered) when determining the model parameters used to predict its E_b value, except for simple models like LD which have only a small number of parameters where potential bias is inherently minimal (we also do not refit the WS4 model parameters). This is especially important when comparing the performance of AI models. The AI model used in this study achieves an RMS on its training data of less than 10 keV, which only means that the model has sufficient capacity to fit the data. In Refs. [24, 25], we quoted an RMS of 130 keV on all of the same nuclei used in this study. That is an unbiased RMS, which required independently training different versions of the AI model such that each E_b value is only considered an unbiased prediction when coming from a model trained without access to that nucleus. Importantly for a multi-task model, when removing $E_b(Z, N)$ this requires also removing from the training data the other properties of nucleus (Z, N). In addition, all the neighboring separation energies must be removed, since $E_b(Z, N)$ can be exactly solved for using the right combination of neighboring separation energies.

Unfortunately, not all AI models quote RMS values in the manner outlined here. AI models that include the training data in the sample used to determine the RMS are (potentially highly) biased. AI models that only consider a single training-testing split and quote an unbiased RMS using only the testing data are only basing their RMS on a small sample of nuclei. This is troublesome since some nuclei are much more difficult to predict than others, and these are unlikely to be included in the RMS sample. Multi-task AI models that are not careful to co-classify all inputs from the same nuclei in their training-testing split, *e.g.* labeling $E_b(Z, N)$ testing but the radius of (Z, N) training, and instead classifying nuclei from each observable independently at random, are also biased. Consequently, this makes precise comparison of the performance of various AI predictions of E_b challenging. We hope that all future works will consider the definition of unbiased RMS described here.

DETAILS ON PHYSICS MODELS

Liquid Drop Model

The famous liquid drop (LD) model, proposed almost a century ago [32, 33], treats the nucleus as a highly dense incompressible fluid. The nuclear binding energy, E_b, within this simple model is given by

$$E_b^{\rm LD} = \alpha_v A - \alpha_s A^{2/3} - \alpha_c \frac{Z(Z-1)}{A^{1/3}} - \alpha_a \frac{(N-Z)^2}{A} + \alpha_p \frac{\delta(Z,N)}{A^{1/2}}\,, \tag{S1}$$

where $A = Z + N$ is the total number of nucleons (protons and neutrons) in the nucleus. The terms in order are referred to as the volume, surface, Coulomb, asymmetry, and pairing terms. For concreteness, when considering only the LD model, we use

$$\delta(Z,N) = \begin{cases} 1 & \text{if } N \text{ and } Z \text{ even} \\ -1 & \text{if } N \text{ and } Z \text{ odd} \\ 0 & \text{otherwise} \end{cases}\,. \tag{S2}$$

Despite its simplicity, after fitting the α_i coefficients to data, the LD predictions achieve a precision that ranges from $\mathcal{O}(\%)$ for moderate nuclei to $\mathcal{O}(0.1\%)$ for large nuclei. Figure S1 shows the residuals of the LD model across the nuclear plane. The RMS for the LD model is roughly 3.0 MeV.

Weizsäcker-Skyrme (WS4)

To the best of our knowledge, version 4 of the Weizsäcker-Skyrme (WS4) model [10] provides the best precision for a non-AI mode. The WS4 model employs a microscopic-macroscopic approach. The microscopic calculations use an

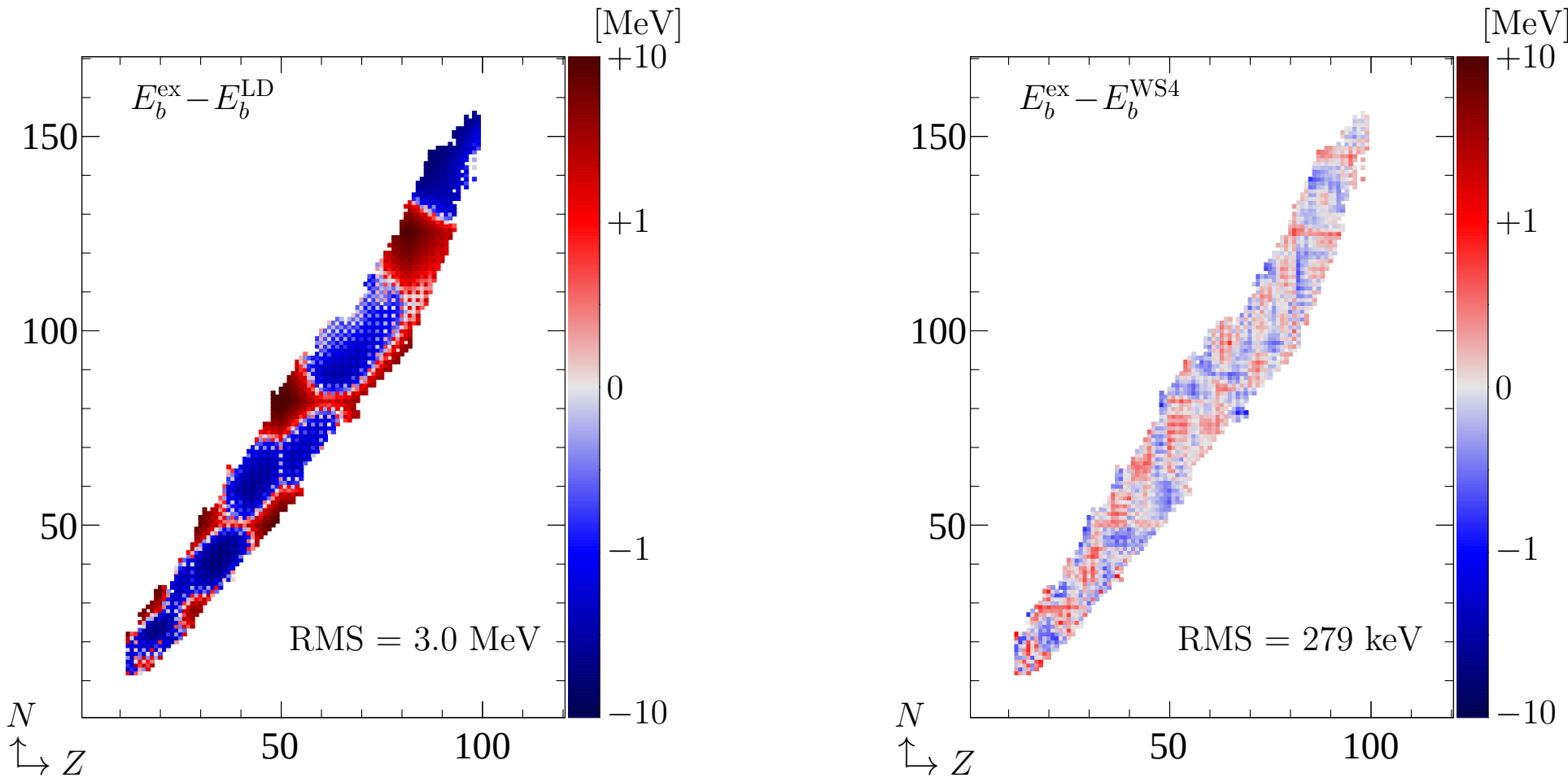

FIG. S1. **Liquid-drop and WS4 residuals show the need for improved models.** Residuals between the (left) liquid-drop (LD) and (right) version 4 of the Weizsäcker-Skyrme (WS4) model nuclear binding-energy, E_b^{LD} and E_b^{WS4}, predictions and the measured experimental values, E_b^{ex}. Note that the color scale is linear on $[-1, 1]$ and logarithmic otherwise. The lack of nuclear shell corrections is evident in the LD residuals.

axially deformed Woods–Saxon potential; see Ref. [10] for details. The macroscopic model used by WS4 applies some corrections to the LD model. The volume and surface terms are unchanged, however the Coulomb term becomes

$$E_b^C = \alpha_C \frac{Z^2}{A^{1/3}} \left(1 - \frac{0.76}{Z^{2/3}}\right) , \tag{S3}$$

where only α_C is determined from a fit to the data. The asymmetry term becomes

$$E_b^a = \alpha_a \left(1 - \frac{\kappa}{A^{1/3}} - \xi \frac{2-|I|}{2+|I|A}\right) I^2 A \left(1 + \kappa_a((I-I_0)^2 - I^4)A^{1/3}\right) , \tag{S4}$$

where α_a, κ, ξ, and κ_a are all parameters of the fit, $I = (N-Z)/A$, and $I_0 = 0.4A/(A+200)$. The version of the pairing term used is

$$E_b^{\mathrm{pair}} = \alpha_{\mathrm{pair}} A^{-1/3} \delta_{np} , \tag{S5}$$

where

$$\delta_{np} = \begin{cases} \frac{17(2-|I|-I^2)}{16} & \text{if } N \text{ and } Z \text{ even} \\ |I| - I^2 & \text{if } N \text{ and } Z \text{ odd} \\ 1 - |I| & \text{if } N \text{ even, } Z \text{ odd, and } N > Z \\ 1 - |I| & \text{if } N \text{ odd, } Z \text{ even, and } N < Z \\ 1 & \text{otherwise} \end{cases} , \tag{S6}$$

and α_{pair} is a parameter to be fit. The final macroscopic term in the WS4 model is the Wigner term, which has the form

$$E_b^W = \alpha_W (e^{|I|} - e^{-|\eta|}) , \tag{S7}$$

where α_W is found from fitting the data, $\eta = (\Delta Z \Delta N)/Z_m$, and ΔZ and ΔN are the number of nuclei between the nucleus of interest and the line defined by $N = 1.37Z + 13.5$ along the Z and N directions, respectively. The quantity Z_m is defined as the hypotenuse of the triangle formed by ΔZ and ΔN. Putting this all together, the predictions of the WS4 model are

$$E_b^{\mathrm{WS4}} = \alpha_v A - \alpha_s A^{2/3} + E_b^C + E_b^a + E_b^{\mathrm{pair}} + E_b^W + E_b^{\mathrm{mic,WS4}} , \tag{S8}$$

where the final term denotes the WS4 microscopic corrections. Therefore, the macroscopic part is a symbolic expression with 9 parameters, while the microscopic part is the output of a simplified many-body quantum-mechanical calculation using a phenomenological potential with 10 parameters determined by fitting the data [10]. Figure S1 shows the residuals of the WS4 model across the nuclear plane. The RMS for the WS4 model is 0.28 MeV.

DETAILS ON THE ARCHITECTURE

To facilitate extracting what knowledge the AI has learned, we study a simplified version of the model in Refs. [24, 25] that is only trained to predict E_b, and thus, only has access to E_b measurements. This AI architecture takes as its only inputs for each nucleus the values of Z and N, which are promoted to learnable 1024-dimensional vectors that are fed into a simple neural network (NN), $F_{\rm nn}$, with learnable parameters $\vec{\theta}$:

$$Z \to \vec{Z}, N \to \vec{N} \Rightarrow E_b = F_{\rm nn}(\vec{Z}, \vec{N}, \vec{\theta})\,. \tag{S9}$$

The first step in the architecture involves concatenating together $\vec{Z}$ and $\vec{N}$ into a single vector with $n = n_Z + n_N$ components. Schematically, the NN is depicted in Fig. S2. The ResBlocks are essentially just $n \to n$ multi-layer-perceptrons plus residual (or skip) connections:

$$\text{ResBlock}(\vec{x}) = \vec{x} + [\vec{x} \to \text{Linear}(n,n) \to \text{ReLU} \to \text{Linear}(n,n) \to \text{ReLU}]\ . \tag{S10}$$

The objective during training is to minimize the following loss function:

$$\mathcal{L} = \sum_i \left(E^{\rm ex}_{b,i} - E^{\rm ai}_{b,i}\right)^2 + \lambda \left[\sum_j Z_j^2 + \sum_k N_k^2 + \sum_\ell \theta_\ell^2\right]\ , \tag{S11}$$

where $E^{\rm ex}_{b,i}$ and $E^{\rm ai}_{b,i}$ are the experimental and AI-predicted E_b values for each nucleus in the training data, and λ is a hyperparameter that controls the balance between prediction quality and model complexity. The sums denoted by j and k run over the components of each of the learned embedding vectors for all allowed Z and N values, respectively; they are not sums over nuclei or over the integer values of Z and N, but rather the 107×1024 components of the 107 $\vec{Z}$ vectors (there are 107 unique values of Z in the nuclear data set) and 167×1024 components of the 167 $\vec{N}$ vectors. The λ hyperparameter is introduced via the Adaptive Moment Estimation (Adam) algorithm, and in our case the variant AdamW [52], which is used during training with learning rate 10^{-4} and weight decay 10^{-1} for over 150,000 epochs.

The 1024-dimensional embeddings are intentionally overcomplete: the physics exposed by the leading PCs occupies only a small number of high-variance directions, while the remaining dimensions give the optimizer enough capacity to find a high-precision solution without imposing a hand-designed low-dimensional ansatz. Within this regularized learned-embedding family, the double-helix geometry is robust across the variations explored in our code and in Refs. [24, 25], including random seed, training-validation splits, learning rate, weight decay, network widths and depths, and training epochs. The baseline runs used here have 1024-dimensional embeddings and two residual blocks; related scripts and earlier studies include four-block/2048-wide models, train fractions from 10–90%, learning rates from 10^{-4} to 10^{-3}, and weight decays from 10^{-8} to 10^{-2}. Ref. [25] also found helical embeddings in a transformer-style fixed-attention setup. This should not be read as a claim that every architecture or activation function must produce the same visible helix; in particular, removing regularization can yield a continuous ordered representation with comparable performance but without the same helical geometry [25].

Finally, we note that while we use the basis (Z, N), it might seem more natural, given Eq. (1), to instead use $(A, Z - N)$. Note that the form of the regularization term in Eq. (S11) is such that both bases give the same penalty term, which means that this alternative basis is just a rotation from our chosen one. For Refs. [24, 25] we chose the (Z, N) basis due to our expectation that the nuclear magic numbers would be special in some way in the embedded structures. Indeed, that is seen in Refs. [24, 25], but not seen here, due to those prior works also training on separation energies where the shell structure is more important.

DETAILS ON THE FORMATION OF A DOUBLE HELIX

Setting the Scale in the Representation Space

The dominant term in Eq. (1) is the volume term, thus $E_b \approx \alpha_v A$. The AI model must be able to build A from $\vec{\mathcal{Z}}$ and $\vec{\mathcal{N}}$. This drives the most important PC components to be

$$\mathcal{Z}_1 \approx \beta Z, \mathcal{N}_1 \approx \beta N \to A \approx (\mathcal{Z}_1 + \mathcal{N}_1)/\beta\,, \tag{S12}$$

where β is a learned scale factor. Addition of $\vec{\mathcal{Z}}$ and $\vec{\mathcal{N}}$ components is trivial to implement in the downstream NN. Therefore, to produce the volume term the NN just needs to store the factor α_v/β in its $\vec{\theta}$ parameters. The

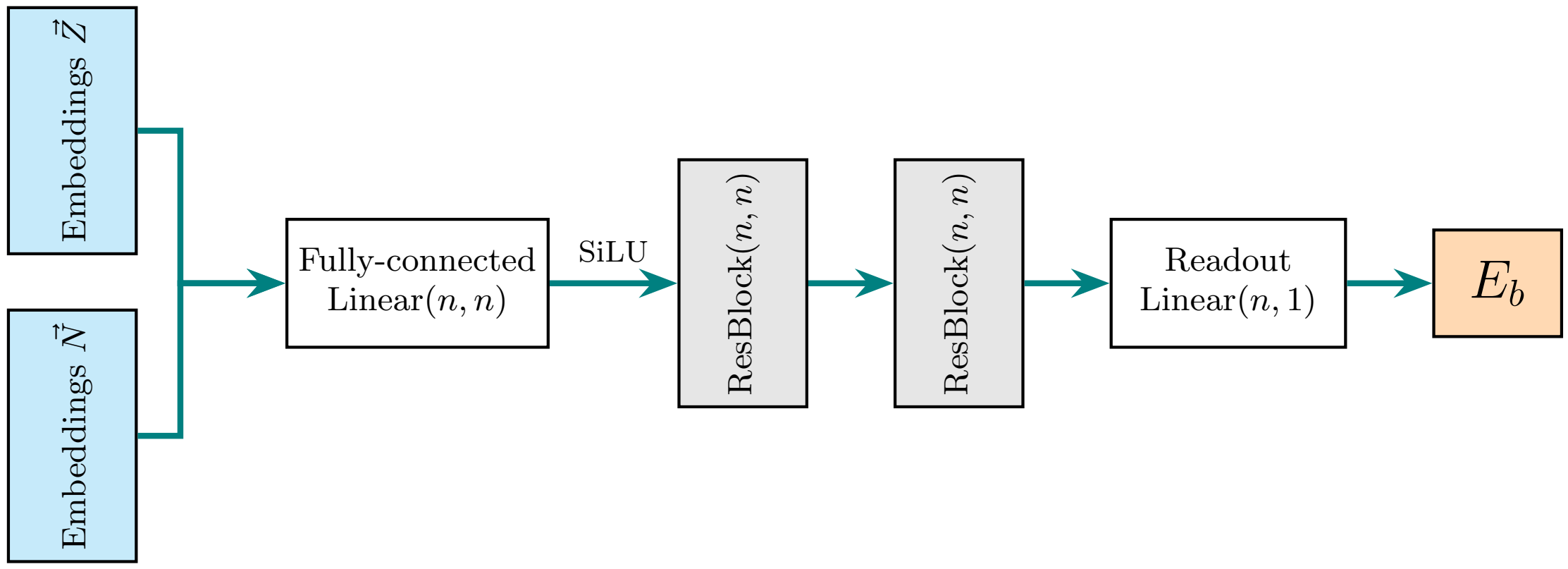

FIG. S2. **Neural-network architecture for binding-energy prediction.** Inputs are embeddings of proton number Z and neutron number N, which are concatenated and then processed through a fully-connected layer and two residual blocks, followed by a readout predicting the binding energy E_b.

regularization term in Eq. (S11) provides a *central force* that attracts all degrees of freedom in the AI model inwards. Naively, this should drive $\beta \to 0$; however, this necessarily results in a contribution from the $\vec{\theta}$ regularization of $\alpha_v/\beta \to \infty$. Consider the simplified case

$$E_b = \alpha_v A, \mathcal{Z}_1 = \beta Z, \mathcal{N}_1 = \beta N, F_{\rm nn}(\vec{Z}, \vec{N}, \vec{\theta}) = \frac{\alpha_v}{\beta}(\mathcal{Z}_1 + \mathcal{N}_1) = \alpha_v A\,. \tag{S13}$$

The scale α_v/β must be encoded as a sum of products of θ parameters, which we will denote generically as $F_{\Sigma\Pi}(\vec{\theta})$, with the constraint that $F_{\Sigma\Pi}(\vec{\theta}) = \alpha_v/\beta$. The form of $F_{\Sigma\Pi}(\vec{\theta})$ depends on the architecture, but the constraint will always be holonomic. In the limit of the best-fit point, *i.e.* where the first term in Eq. (S11) vanishes, the loss at the minimum satisfies

$$\vec{\nabla}\mathcal{L} = \Lambda \vec{\nabla}\left(F_{\Sigma\Pi}(\vec{\theta}) - \frac{\alpha_v}{\beta}\right), \tag{S14}$$

where Λ denotes a Lagrange multiplier and the gradient is with respect to all learnable parameters of the AI model. The optimal loss in this case satisfies

$$\mathcal{L} = \lambda\left[\beta^2(s_Z + s_N) + \sum_\ell \theta_\ell^2\right], \qquad s_Z = \sum_Z Z^2, \qquad s_N = \sum_N N^2\,, \tag{S15}$$

where $s_{Z,N}$ are geometric sums of the (unique) values of Z and N found in the training data set, hence $s_{Z,N}$ are constants that do not vary during training. This reduction to unique Z and N values is a feature of the simplified volume-term construction studied here, where sparsely embedding coordinates proportional to the integer labels is sufficient to fully specify the target function. The value of β that minimizes the loss is thus

$$\beta = \left[\frac{\Lambda \alpha_v}{2\lambda(s_Z + s_N)}\right]^{1/3}, \tag{S16}$$

where the value of Λ depends on $F_{\Sigma\Pi}(\vec{\theta})$, and in general $\Lambda \propto \beta^\gamma$ for some power γ. (For our architecture, the optimal solution is either $\gamma = 0$ or $1/2$ depending on the values of α_v, λ, and $(\sigma_Z + \sigma_N)$.) We can see that the constraint of obtaining the natural scale α_v results in determining a (non-zero) scale for the PCs, *i.e.*, $\beta \neq 0$. We confirm this by taking a frozen model, *i.e.* a model where the neurons of all downstream layers are kept constant, and rescale the first PC components, which as expected gives

$$\mathcal{Z}_1 \to (1 \pm \delta)\mathcal{Z}_1, \mathcal{N}_1 \to (1 \pm \delta)\mathcal{N}_1 \Rightarrow E_b \to (1 \pm \delta)\alpha_v A\,. \tag{S17}$$

Therefore, the compressive regularization pressure is counter-balanced by the goodness-of-fit term in $\mathcal{L}$, which effectively produces a *repulsive force* when the embedding vectors get too close along the first PC axis (similar to the Van der Waals force in DNA). The scale β is set by the natural scale α_v, along with details about the architecture and training data.

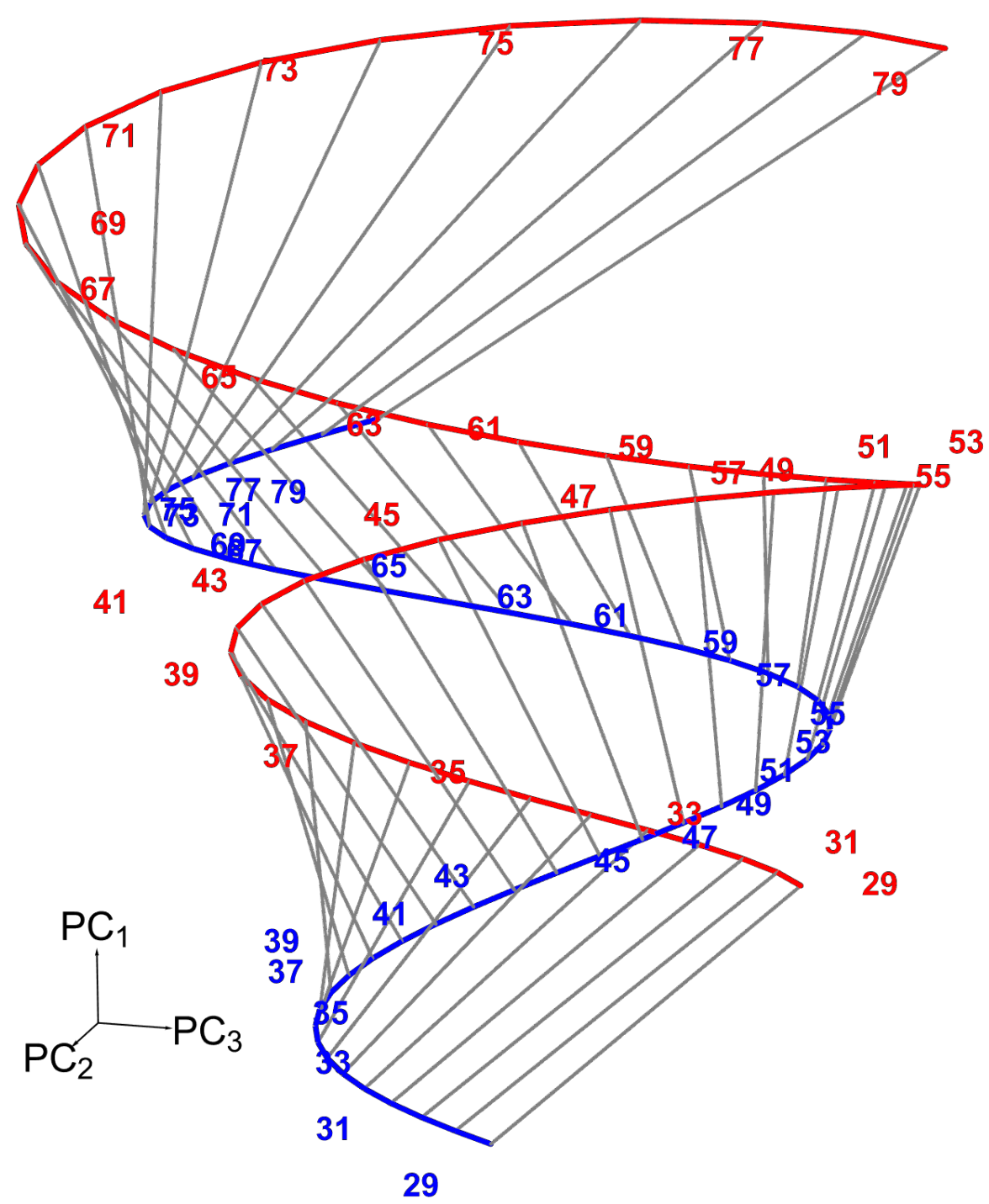

FIG. S3. **Isospin-symmetric training data produce a symmetric double helix.** The three most important principal components (PCs) of the internal representations learned by our AI model for (red) Z and (blue) N when trained on simulated isospin-symmetric data. The links connect the values of Z and N found in the most stable nucleus of each isotopic chain, which is $Z = N$ for this simulated data. The curves show fits of helices to the Z and N representations. Note that only the odd points are labeled to avoid clutter.

Forming Helices

The same reasoning that prevents the first PC from collapsing under the regularization pressure also applies to the second and third PC dimensions shown in Fig. 1; we will show this later in this section. However, this does not explain the origins of the helix structure. Naively, there are no cyclic or oscillatory terms in Eq. (1), making it surprising that the second and third most important PCs would form an oscillatory structure.

To understand why an oscillatory structure is optimal here, we next consider the second most important term in Eq. (1), the asymmetry term, which contains a factor of $(Z-N)^2$. This term is invariant under transformations of the form $Z \to Z+\gamma, N \to N+\gamma$, which correspond to translations along the $\hat{\mathcal{Z}}_1$ and $\hat{\mathcal{N}}_1$ axes (which are nearly aligned) in Fig. 1. What matters when computing $(Z-N)^2$ is only the relative difference in Z and N; thus, the same solution can be repeated all along the first PC axis, which is desirable due to the regularization pressure. In principle our AI model could learn to store $(Z-N)^2$ for each (Z,N) pair, but the regularization pressure forces it to instead find a more efficient solution, and the helix is the most efficient option.

As in the previous subsection, it is illustrative to consider a simplified scenario. We generate isospin-symmetric training data using

$$E_b = \alpha_v A - \alpha_a \frac{(Z-N)^2}{A}\,, \tag{S18}$$

and enforce that the observed nuclei are symmetric in the sense that for any generated nucleus (Z,N) the mirror nucleus (N,Z) is also generated. Figure S3 shows that a nearly isospin-symmetric double helix is formed in this scenario. Given the dominance of the volume term, we again find the relationship from Eq. (S12). The focus here will be on PC dimensions 2 and 3, namely the second and third most important PCs.

In the limit of a perfect double helix in the first 3 components of both $\vec{\mathcal{Z}}$ and $\vec{\mathcal{N}}$, we first note that the number of

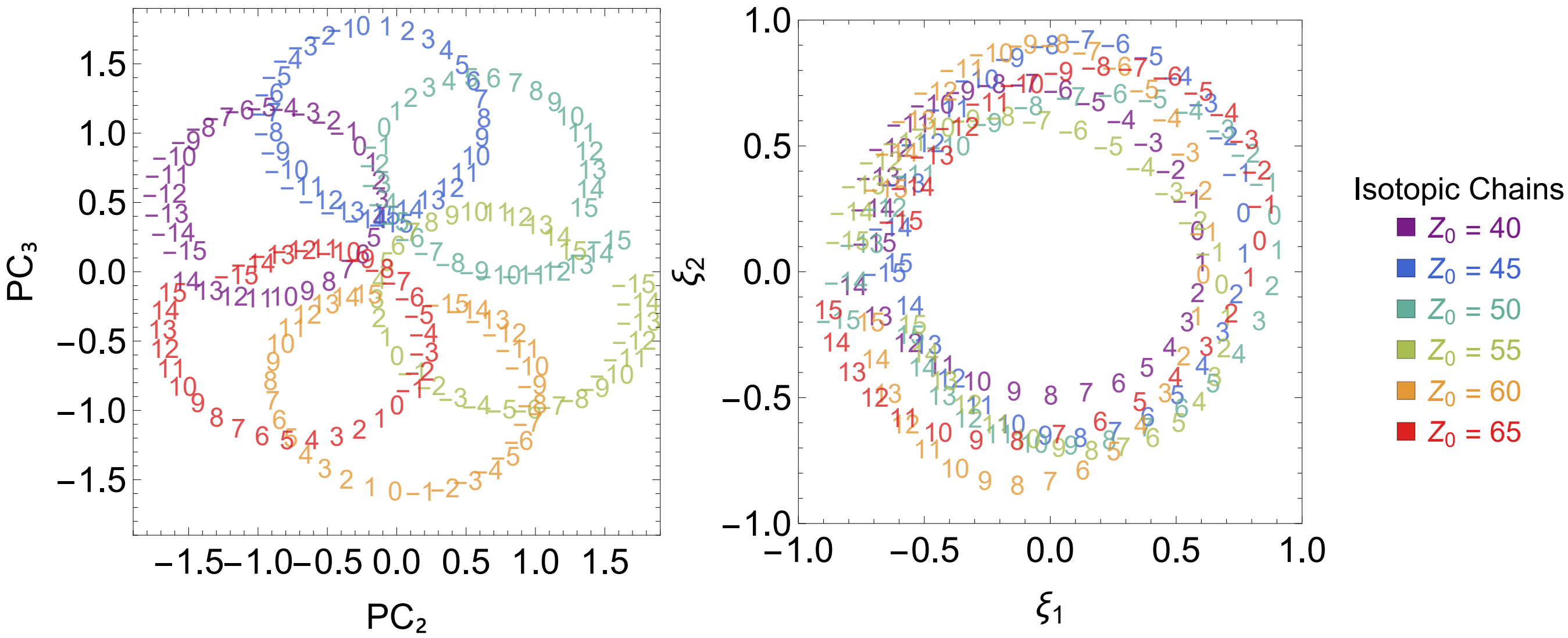

FIG. S4. **The helical embedding acts as a clock for proton–neutron imbalance.** Left: Embedding vector components $(\mathcal{N}_2 - \mathcal{Z}_2, \mathcal{N}_3 - \mathcal{Z}_3)$ linking each point Z_0 on the Z helix with the points $[N_0 - 15, N_0 + 15]$ with $N_0 = Z_0$ on the N helix. The numbers for each labeled point denote $N - N_0 = N - Z_0$. Right: Transformation of the points in the left panel according to Eq. (S20). Here, $\mathcal{Z}_i$ and $\mathcal{N}_i$ denote the ith principal components of the proton number Z and neutron number N embedding vectors, respectively.

isotopes (equivalently isotones), n_I, is related to the angular frequency of points along the helix by

$$\omega \approx 2\pi / n_I \,. \tag{S19}$$

Next, consider the following vector in the PC_2-PC_3 plane:

$$\vec{\xi} = \begin{pmatrix} \mathcal{Z}_2 \mathcal{N}_2 - \mathcal{Z}_3 \mathcal{N}_3 \\ \mathcal{Z}_2 \mathcal{N}_3 + \mathcal{Z}_3 \mathcal{N}_2 \end{pmatrix} . \tag{S20}$$

Figure S4 shows that, in this isospin-symmetric scenario, for any isotope (considering all $\vec{\xi}$ for a fixed Z_0)

$$\vec{\xi}(Z_0, N) \propto \begin{pmatrix} \cos\left[(Z_0 - N)\omega\right] \\ \sin\left[(Z_0 - N)\omega\right] \end{pmatrix} , \tag{S21}$$

where a similar mirror relationship exists for isotones. (This relationship is not exact due to effects such as the $\mathcal{Z}_1$ and $\mathcal{N}_1$ axes not being truly parallel, and some leakage of information into the lesser PC components.) Evidently, the simple construction Eq. (S20) encodes the value of $(Z - N)$ needed to build the asymmetry term.

A transformer-based model could easily construct $\vec{\xi}$ using the dot product operator provided by the attention mechanism. (Notice that Eq. (S20) involves products of components of the embedding vectors.) However, our model does not use attention. It is straightforward though to build up $\vec{\xi}$ instead using only sums of embedding components and nonlinearities in the NN. See Appendix A of Ref. [53] for a derivation showing how to obtain $\vec{\xi}$ from only sums and nonlinearities. Of note, whereas $\vec{\xi}$ as defined in Eq. (S20) is proportional to the radius of the helices squared, ρ^2, since the components in Eq. (S20) involve products of $\mathcal{Z}_{2,3}$ and $\mathcal{N}_{2,3}$ components, for the case where attention is not used this proportionality changes to $|\vec{\xi}| \propto \rho$. As in the previous subsection, the regularization pressure naively drives $\rho \to 0$, but again the need to predict the scale of the asymmetry term, driven by the constant α_a, leads to an optimal solution where α_a is encoded as ρ multiplied by a sum of products of θ parameters. We confirmed this by taking a frozen model and rescaling ρ, which gives

$$\rho \to \beta\rho \Rightarrow E_b \to \alpha_v A - \beta \alpha_a \frac{(Z - N)^2}{A} \,, \tag{S22}$$

as expected for this architecture.

Finally, we note that the electromagnetic interactions of protons breaks the symmetry between Z and N, which is why the double helix in Fig. 1 is itself not symmetric. For example, the most stable nuclei in nature do not have $Z = N$; the Coulomb term in Eq. (1) requires many more neutrons than protons in large stable nuclei. The nuclear shell structure is also an important factor in determining which nucleus is the most stable for each isotopic chain. These result in the bond analogs in Fig. 1 not being parallel like they are in Fig. S3 (especially close to magic nuclei).

PHYSICS ORIGINS OF JAFFE FACTORIZATION

The Garvey-Kelson (GK) relations are patterns found in nuclear data, where combinations of E_b from a set of nuclei approximately cancel almost anywhere in the nuclear plane [34]. For example, if $Z > N$,

$$E_b(Z{+}2, N{-}2) - E_b(Z{+}2, N{-}1) - E_b(Z{+}1, N{-}2) + E_b(Z, N{-}1) + E_b(Z{+}1, N) - E_b(Z, N) \approx 0\,, \tag{S23}$$

whereas if $Z < N$,

$$E_b(Z{-}2, N{+}2) - E_b(Z{-}1, N{+}2) - E_b(Z{-}2, N{+}1) + E_b(Z{-}1, N) + E_b(Z, N{+}1) - E_b(Z, N) \approx 0\,. \tag{S24}$$

Other GK relations also approximately hold, such as

$$E_b(Z, N{+}2) - E_b(Z{-}2, N) + E_b(Z{-}2, N{+}1) - E_b(Z{-}1, N{+}2) + E_b(Z{-}1, N) - E_b(Z, N{+}1) \approx 0\,. \tag{S25}$$

The GK relations arise because local protonic and neutronic energy increments do not vary much in a small region of the nuclear plane. The *energy levels* in the following argument should be understood as effective local protonic and neutronic increments in a small patch of the nuclear plane, not as a microscopic claim that the absolute binding energy is a literal global sum of independent single-particle energies. Therefore, in any such small region around a reference point (Z_0, N_0) we can write

$$E_b(Z_0 + \delta Z, N_0 + \delta N) \approx \sum_{i=1}^{Z_0+\delta Z} E_i^p(Z_0, N_0) + \sum_{j=1}^{N_0+\delta N} E_j^n(Z_0, N_0)\,, \tag{S26}$$

where macroscopic terms are approximately constant over the small region and can thus be absorbed into the definitions of E_i^p and E_j^n (since most of the terms in each sum are shared by all nuclei in the small region). Notice that each local increment appears equally with a plus and minus sign in the GK relations; therefore, plugging Eq. (S26) into any GK relation gives zero under the assumption that these increments are constant within the region considered. In the limit that the GK relation holds exactly, we thus have

$$E_b(Z_0 + \delta Z, N_0 + \delta N) \Rightarrow F_Z(Z_0 + \delta Z) + F_N(N_0 + \delta N)\,, \tag{S27}$$

which is Jaffe factorization. Any contribution to E_b that varies slowly over the nuclear plane, *e.g.*, macroscopic terms like Eq. (1), approximately cancels in the GK relations, but not exactly, hence this factorization approximation only holds in small local regions of the nuclear plane. Finally, note that in Ref. [34] two non-factorizable terms also appear, including a constant multiplied by ZN. Following the derivation presented here, that constant only need not vary over the small region around (Z, N), *e.g.*, it could be chosen to be $-2\alpha_a/A$ and the ZN term could thus be absorbed into the asymmetry term from Eq. 1. The other non-factorized term presented in Ref. [34] similarly can be absorbed into the pairing term. Clearly we can also factor out any constants from $F_Z + F_N$, and absorb them into the macroscopic model as well as other slowly varying contributions to E_b. This is why we only focus on the factorized expression.

PENULTIMATE-LAYER PRINCIPLE COMPONENTS & OUR SYMBOLIC MODEL

Figure 2 shows the 6 most important PCs of the penultimate NN layer, where the sum of all such PCs is E_b^{ai}. The first PC is dominated by the volume term. In fact, the first two PCs when summed are well approximated by Eq. (1) with the exception of what are typically called shell terms (more on these below). The third PC visually has an approximate linear dependence on $Z - N$, and we find it cannot be fit well without the Wigner term described in the main text. The lesser PCs all exhibit Jaffe factorization to a high degree, such that the RMS from fitting the sum of all lesser PCs to Eq. (S26) is around 0.1 MeV. Note, however, that the fifth PC also has a strong overlap with the pairing term (which itself approximately obeys Jaffe factorization).

As an explicit example below the scale of the first six displayed PCs, Fig. S6 shows $\mathcal{E}_{b,17}$, whose standard deviation is 95 keV. A fit of the form $F_Z(Z) + F_N(N)$ leaves a residual RMS of 4 keV, showing that the factorized structure persists at the 100 keV scale relevant to the AI model precision.

We build the macroscopic portion of our symbolic model by fitting the sum of the first 3 PCs, which are approximately smooth functions. This base model largely follows the one used by WS4, but with a few modifications. In addition to the terms defined above, our model includes corrections near the double-magic nuclei $^{56}_{28}$Ni, $^{78}_{28}$Ni, $^{100}_{50}$Sn, $^{132}_{50}$Sn, and $^{208}_{82}$Pb of the form

$$E_b^{\mathrm{DM}} = \alpha_{\mathrm{DM}} e^{-\beta_{\mathrm{DM}}\Delta}\,, \tag{S28}$$

where Δ is the Euclidean distance to the double-magic nucleus. We also include a microscopic portion that consists of the Jaffe relations as described above by Eq. (5), since even the first 3 PCs do have discrete Jaffe-like terms built into them (mostly describing nuclear shell structure). To be explicit, the full model is

$$E_b^{\mathrm{sym}} = \alpha_v A - \alpha_s A^{2/3} + E_b^C + E_b^a + E_b^{\mathrm{pair}} + \alpha_W \frac{|Z-N|}{A} + E_b^{\mathrm{DM}} + E_b^{\mathrm{mic,Jaffe}}\,. \tag{S29}$$

The volume and surface terms are unchanged from Eq. (1). The Coulomb, asymmetry, and pairing terms are from Eqs. (S3)–(S5), and the BW2 Wigner term [35] is as described in the main text. These are combined with 249 constants, one for each N and Z value for the global Jaffe corrections. As discussed in the main text, the model is improved by replacing the global Jaffe terms with local ones; these are described in the following sections.

Some care must be taken when fitting this model since the global Jaffe corrections have some degeneracy with the macroscopic terms and each other. For example, the volume term can be factorized according to Eq. (5), and thus can be absorbed into the $E_b^{\mathrm{mic,Jaffe}}$ term, setting $\alpha_V = 0$. While this results in equivalent predictions, it reduces interpretability and removes physical meaning from the model. Similarly, the Jaffe corrections can be degenerate with each other, *e.g.* by adding a constant term to F_Z and subtracting it from F_N. To deal with these two forms of degeneracy, we include Gaussian penalties around the parameters in the first 6 terms of Eq. S29 and L1 regularization on the microscopic parameters in the loss for the fit. The full loss to be minimized is thus

$$\mathcal{L} = \sum_i \left(E_{b,i}^{\mathrm{ex}} - E_{b,i}^{\mathrm{sym}}\right)^2 + \lambda_G \sum_i \left(\frac{\alpha_i^{\mathrm{fit}} - \bar{\alpha}_i}{\bar{\alpha}_i}\right)^2 + \lambda_R \sum_i |\theta_i^J|\,, \tag{S30}$$

where $E_{b,i}^{\mathrm{ex}}$ and $E_{b,i}^{\mathrm{sym}}$ are the experimental and predicted E_b values for each nucleus, λ_G and λ_R are hyperparameters which control the strictness of the Gaussian and L1 constraints, α_i^{fit} and $\bar{\alpha}_i$ are the parameters fit in the full model or only macroscopic version, and θ_i^J is the set of microscopic corrections.

Figure S7 shows the results of fitting the first 3 PCs to Eq. (S29). The first 3 PCs are described well, and the shell structure contained in them is clearly visible in the microscopic part of the function. Figure S8 shows the residuals from fitting Eq. (S29) to the data. The RMS is 0.37 MeV using the global Jaffe terms. Given the simplicity of this model, its accuracy is impressive. Using the local Jaffe corrections reduces the RMS substantially as well, as described below and in the main text. Local Jaffe corrections are discussed in detail in the next section.

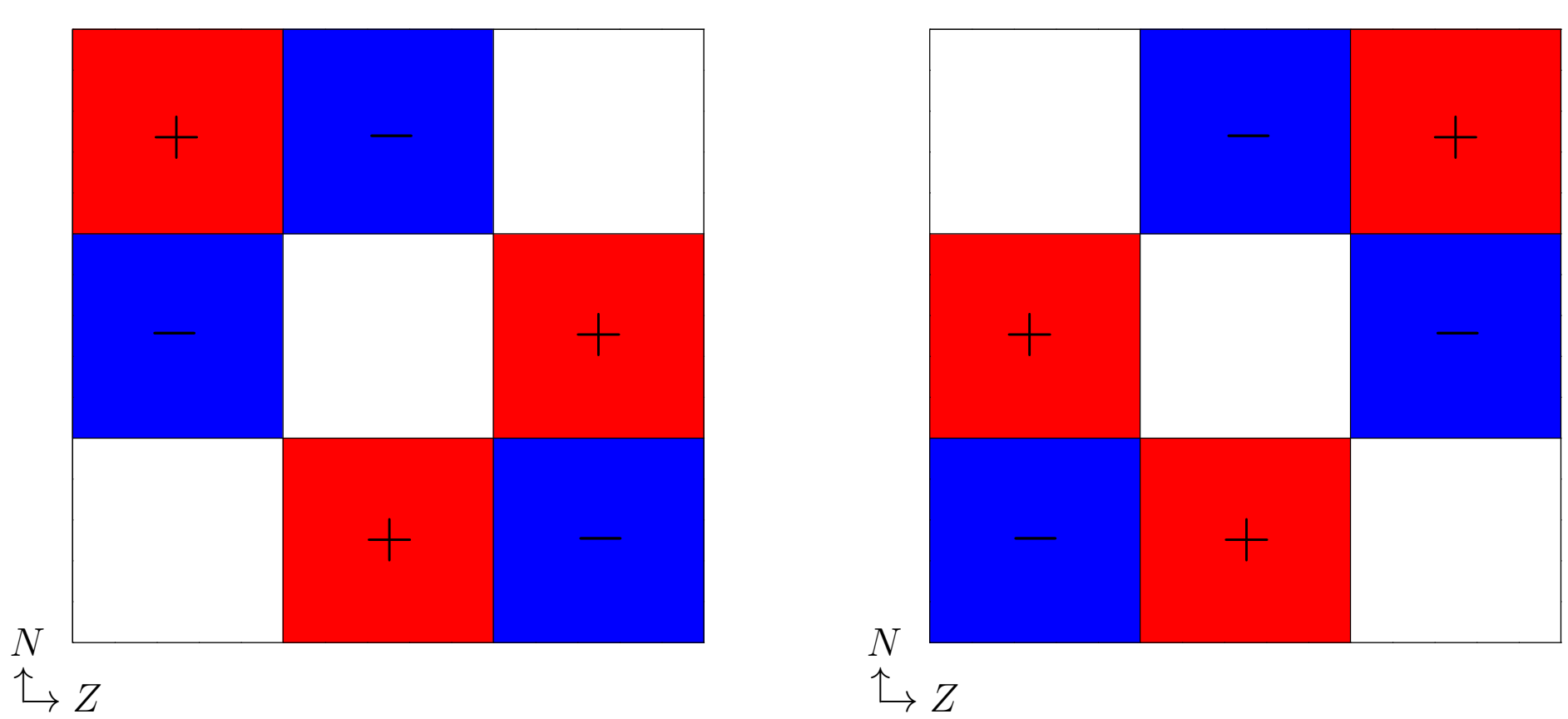

FIG. S5. **Garvey–Kelson relations cancel local protonic and neutronic increments.** Examples of GK relations in the (Z, N) plane for Eqs. (S24) and (S25). The relative sign (addition or subtraction) of the contribution of each nuclear binding energy, E_b, value is labeled on the panel (also colored red for addition, blue for subtraction).

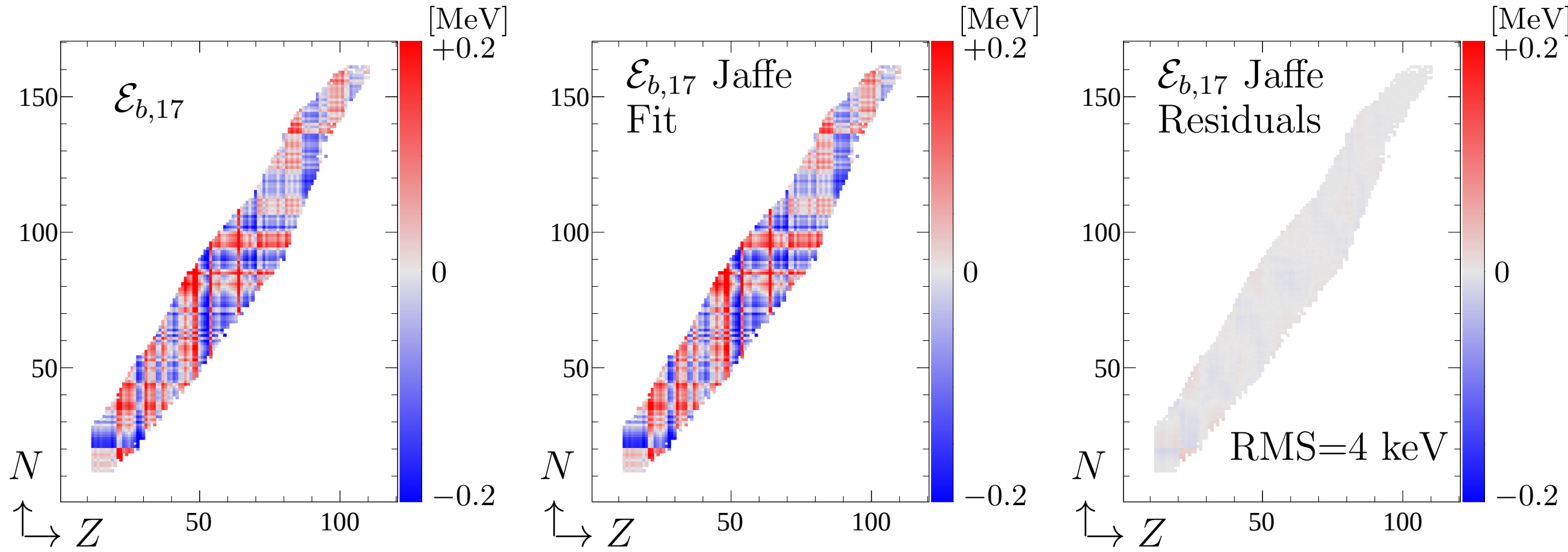

FIG. S6. **Representative higher-order AI binding-energy principal component obeys Jaffe factorization at the keV scale.** Left: $\mathcal{E}_{b,17}$, a lower-variance binding energy PC with a 95 keV standard deviation. Middle: Best fit of $\mathcal{E}_{b,17}$ to the Jaffe factorized form $F_Z(Z) + F_N(N)$, where F_Z and F_N are functions of only proton number Z and neutron number N, respectively. Right: Residuals of this fit with an RMS of 4 keV.

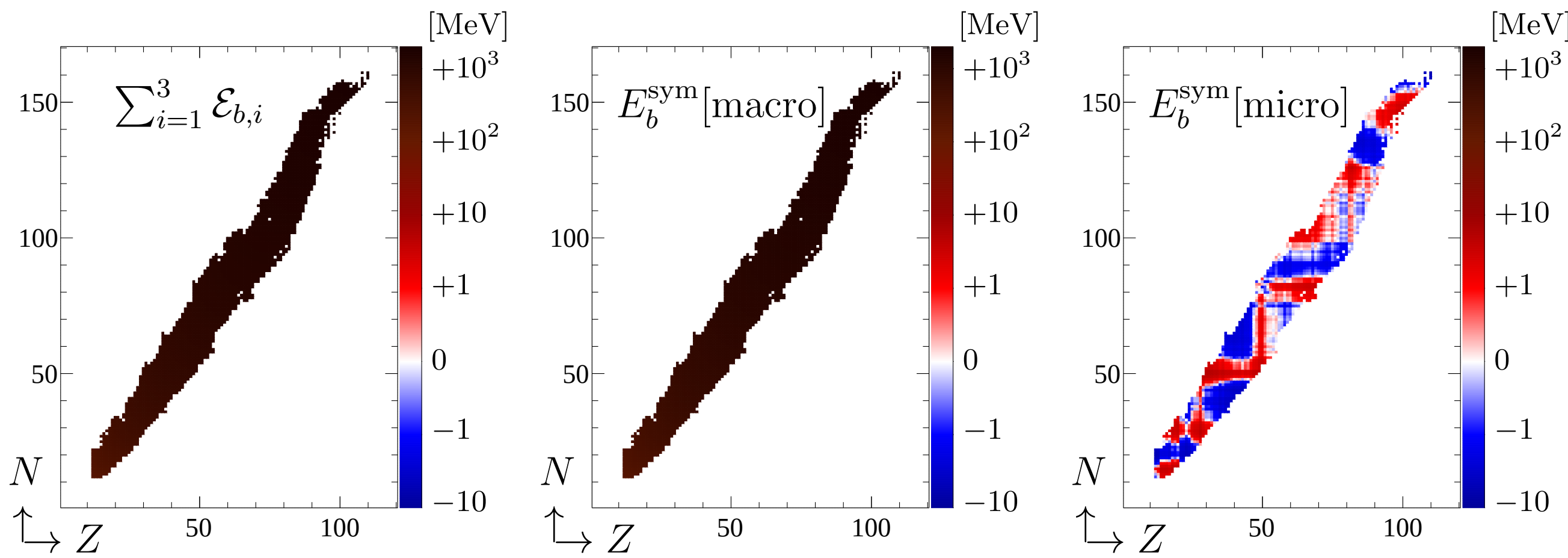

FIG. S7. **Symbolic macroscopic and Jaffe-factorized terms reproduce the leading AI components.** Left: Sum of the 3 most important principal components (PCs) in the penultimate neural network layer, denoted by $\mathcal{E}_{b,i}$. Middle: Smooth symbolic (sym) macroscopic model for binding energy, E_b, obtained by fitting the data shown in the left panel. Right: Microscopic model obtained from the same fit using simple factorized $F_Z(Z)+F_N(N)$ terms, where Z and N denote the proton and neutron number, respectively. The predominant features observed in these microscopic terms are the shell structure near nuclear magic numbers.

JAFFE FACTORIZATION & LOCAL CORRECTIONS

A common approach to using AI to improve the precision of E_b predictions is to start from the WS4 model and learn how to interpolate and extrapolate corrections for each nucleus based on the residuals of its neighboring nuclei. A simplified non-AI version of this approach is to use nearest-neighbor and possibly next-to-nearest-neighbor nuclei to derive the correction as follows:

$$E_b = E_b^{\mathrm{WS4}} + \sum_i \frac{\left(E_{b,i}^{\mathrm{ex}} - E_{b,i}^{\mathrm{WS4}}\right)}{\sqrt{(Z-Z_i)^2 + (N-N_i)^2}} \cdot \left[\sum_i \left((Z-Z_i)^2 + (N-N_i)^2\right)^{-1/2}\right]^{-1}, \tag{S31}$$

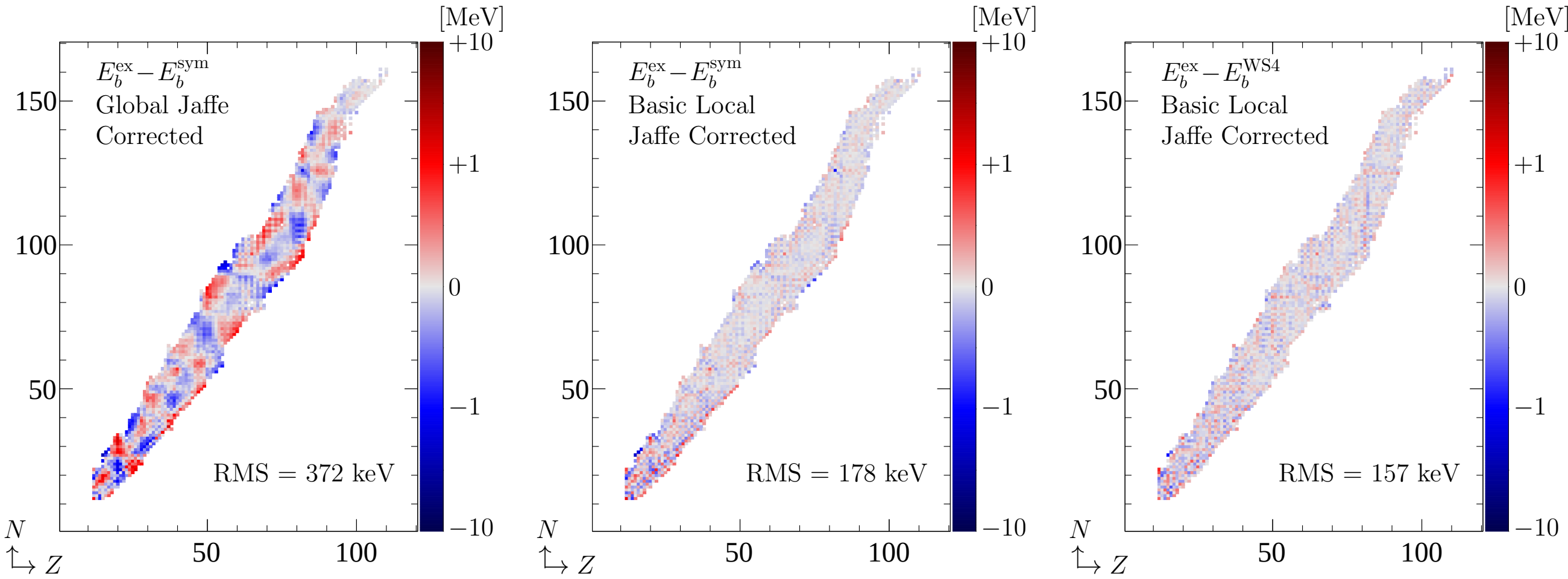

FIG. S8. **Local Jaffe corrections substantially reduce symbolic-model and WS4 residuals.** Residuals between the experimental measurements and our symbolic model, $E_b^{\rm ex} - E_b^{\rm sym}$, with (left) global and (middle) basic local Jaffe corrections, along with (right) the WS4 model with basic local Jaffe corrections. Here, global Jaffe corrections refer to fitting the factorized form $F_Z(Z)+F_N(N)$ across the entire (Z, N) nuclear plane, whereas basic local Jaffe refers to corrections that use only measured nearest and next-to-nearest nuclei with the same Z or the same N, excluding the target nucleus. Note that the color scale is linear on $[-1, 1]$ and logarithmic otherwise.

where the sum runs over a set of neighboring nuclei. Kernels other than the inverse distance give nearly identical results, hence only the inverse-distance kernel is shown here. Applying such corrections gives the following results.

No corrections: The WS4 model without any neighbor-based corrections has an RMS of 279 keV.

All nearest neighbors: Using all nuclei with $|Z - Z_i| \leq 1$, $|N - N_i| \leq 1$, and $(Z, N) \neq (Z_i, N_i)$ to derive the local correction reduces the RMS to 207 keV.

Only isoto(p,n)ic nearest neighbors: Using only nuclei with either $Z = Z_i$ and $|N - N_i| = 1$ or $N = N_i$ and $|Z - Z_i| = 1$ to derive the local correction, which is half the nuclei from the previous case, does even better, reducing the RMS to 175 keV.

Only non-isoto(p,n)ic nearest neighbors: Conversely, using nuclei with $|Z - Z_i| = 1$ and $|N - N_i| = 1$ to derive the local correction does much worse than the previous case, despite using the same number of nuclei, yielding an RMS of 277 keV, consistent with the uncorrected WS4 model.

Only isoto(p,n)ic next-to-nearest neighbors: Using nuclei with either $Z = Z_i$ and $|N - N_i| = 2$ or $N = N_i$ and $|Z - Z_i| = 2$ to derive the local correction gives an RMS of 186 keV. Even though these nuclei are all farther away than the non-isoto(p,n)ic nearest neighbors, they provide a much better correction.

Only isoto(p,n)ic (next-to-)nearest neighbors: Using nuclei with either $Z = Z_i$ and $1 \leq |N - N_i| \leq 2$ or $N = N_i$ and $1 \leq |Z - Z_i| \leq 2$ to derive the local correction gives an RMS of 160 keV. This value is an improvement over the much more complicated approach using radial basis vectors in Ref. [54].

Only non-isoto(p,n)ic (next-to-)nearest neighbors: Conversely, nuclei with with $1 \leq |Z - Z_i| \leq 2$ and $1 \leq |N - N_i| \leq 2$ to derive the local correction does much worse than the previous case, despite using twice as many nuclei, producing an RMS of 242 keV.

These results demonstrate the efficacy of Jaffe factorization [34] applied to local corrections, as illustrated in Fig. 3. Naively, the closer in the nuclear plane a given nucleus is to the target nucleus, one would expect it gives more information about how to correct the WS4 prediction. However, this is not the case. The isoto(p,n)ic neighbors provide extremely valuable information to use in deriving corrections to WS4, whereas the non-isoto(p,n)ic neighbors do not (directly, see following section). The problem is not purely geometric in the nuclear plane due to the quantum-mechanical nature of nuclei (see discussion in the *Physics Origins of Jaffe Factorization* section).

We previously showed that Jaffe factorization arises as a local approximation based on the fact that the single-nucleon energy levels do not vary much within a small region of the nuclear plane. Equation (S27) is a direct consequence of this approximation, and using it to derive the value of $E_b(Z, N)$ from only its isoto(p,n)ic nearest

neighbors gives

$$
\begin{aligned}
E_b(Z,N) &\approx \frac{1}{4}\left[F_Z(Z)+F_N(N-1)+F_Z(Z)+F_N(N+1)+F_Z(Z-1)+F_N(N)+F_Z(Z+1)+F_N(N)\right] \\
&= \frac{1}{2}\left[F_Z(Z)+F_N(N)\right]+\frac{1}{2}\left[\frac{F_Z(Z-1)+F_Z(Z+1)}{2}+\frac{F_N(N-1)+F_N(N+1)}{2}\right] \\
&= F_Z(Z)+F_N(N)+\delta_Z+\delta_N\,.
\end{aligned} \tag{S32}
$$

The approximation made to get to the third line is exact if F_Z and F_N are linear functions, with the errors induced by nonlinearities in $F_{Z,N}$ denoted $\delta_{Z,N}$. Therefore, this local Jaffe interpolation is valid up to non-linear corrections required when changing Z or N by one unit. Using only the non-isoto(p,n)ic neighbors instead gives

$$
\begin{aligned}
E_b(Z,N) &\approx \frac{1}{4}\left[F_Z(Z-1)+F_N(N-1)+F_Z(Z-1)+F_N(N+1)+F_Z(Z+1)+F_N(N-1)+F_Z(Z+1)+F_N(N+1)\right] \\
&= \frac{F_Z(Z-1)+F_Z(Z+1)}{2}+\frac{F_N(N-1)+F_N(N+1)}{2} \\
&= F_Z(Z)+F_N(N)+2\left(\delta_Z+\delta_N\right)\,.
\end{aligned} \tag{S33}
$$

Notice that this approximation does not contain $F_Z(Z)+F_N(N)$ directly like the previous one does—and is twice as sensitive to non-linear errors. The isoto(p,n)ic neighbors share the same filled proton or neutron energy levels as the target nucleus, with only one vacant or extra filled neutron or proton energy level; whereas the non-isoto(p,n)ic neighbors always have a difference in occupancy of energy levels for both the protons and neutrons. This explains why Jaffe corrections vastly outperform distance-based methods in deriving local corrections for nuclear masses.

The physics of local Jaffe corrections can be illustrated by redoing the derivations of the previous paragraph starting instead from Eq. (S26). For the isoto(p,n)ic nearest neighbors this gives

$$
\begin{aligned}
E_b(Z,N) &\approx \frac{1}{4}\left[2\sum_{i=1}^{Z}E_i^p+\sum_{j=1}^{N-1}E_j^n+\sum_{j=1}^{N+1}E_j^n+\sum_{i=1}^{Z-1}E_i^p+2\sum_{j=1}^{N}E_j^n+\sum_{i=1}^{Z+1}E_i^p\right] \\
&= \sum_{i=1}^{Z}E_i^p+\sum_{j=1}^{N}E_j^n+\frac{1}{4}\left[(E_{Z+1}^p-E_Z^p)+(E_{N+1}^n-E_N^n)\right]\,.
\end{aligned} \tag{S34}
$$

We see that the errors due to nonlinearities, $\delta_{Z,N}$, arise from differences in the single-nucleon energy levels. Repeating this calculation for the non-isoto(p,n)ic neighbors gives the same result but with $1/4 \to 1/2$ in the last term, as expected since as already shown the $\times$ interpolation is twice as sensitive to nonlinearities as the $+$ interpolation.

Finally, we find a small improvement by not using odd-odd $Z=N$ neighbors when deriving the local Jaffe corrections. In addition, we do not attempt to correct odd-odd $Z=N$ nuclei. These improvements, *e.g.*,reduce the RMS from 160 to 157 keV for the (next-to-)nearest isoto(p,n)ic neighbors. These small modifications are included in Fig. S8, though the impact on the RMS is small.

IMPROVING ON BASIC LOCAL JAFFE CORRECTIONS

Consider the up to 24 nuclei made up of all observed nearest and next-to-nearest neighbors of a target nucleus (Z,N). The isotopic neighbors used in the local Jaffe corrections from the previous section induce some error due to the implicit assumption that the discrepancy between WS4 and data is only due to the protonic component of E_b. Similarly, the isotonic neighbors introduce an error due to the assumption that the discrepancy is only due to the neutronic component. We can instead take each E_b value to locally be

$$
E_b(Z,N) \approx E_b^{\rm WS4}(Z,N)+\alpha_Z+\alpha_N\,, \tag{S35}
$$

where α_Z and α_N are corrections to the protonic and neutronic WS4 energy levels near the nucleus (Z,N). Previously, we obtained α_Z and α_N using the distance-weighted averages of the isotopic and isotonic neighbors (local Jaffe corrections as in Fig. 3). We can improve on this by instead solving for these corrections by minimizing

$$
\chi^2=\sum_{Z',N'}\mathbf{1}[(Z',N')\neq(Z,N)]\left(E_b^{\rm ex}-E_b^{\rm WS4}-\alpha_{Z'}-\alpha_{N'}\right)^2\,,\ |Z-Z'|\leq 2\,,\ |N-N'|\leq 2\,, \tag{S36}
$$

Number (Next-to-)Nearest Neighbors	WS4 RMS	GK Corrected RMS	Improved Local Jaffe Corrected RMS
20–24	268 keV	93 keV	94 keV
11–19	300 keV	161 keV	160 keV
1–10	391 keV	293 keV	207 keV

TABLE S1. Performance of the WS4 model without (middle) and with (right) local Jaffe corrections for nuclei categorized by how many of their (next-to-)nearest neighbors are included in the data sample. If all of the (next-to-)nearest neighbors are observed, the value is 24. As expected the performance of the WS4 model, along with both Garvey-Kelson (GK) and our local Jaffe corrections degrades when moving towards the edges of the observed nuclear plane; however, even when most of the neighbors are not observed, the local Jaffe procedure still drastically improves the precision of the nuclear mass predictions. In addition, our Local Jaffe corrections outperform the GK-based corrections for nuclei with few observed neighbors.

where **1** is an indicator function that excludes the target nucleus from the sum (since its E_b value cannot be used in its own prediction), and of course, also excludes all unobserved nuclei. This approach allows us to simultaneously correct the neutronic (protonic) component for isotopic (isotonic) neighbors while using them to obtain the protonic (neutronic) correction. Note that as in the basic local Jaffe corrections described previously, we find a small improvement by excluding neighbors on the opposite side of the $Z = N$ line.

Applying this procedure—except for $Z = N$ odd-odd nuclei, and nuclei with less than 7 total and 3 isoto(p,n)ic measured neighbors (of the 24 possible), where we instead apply the basic Jaffe procedure from the previous section—to the WS4 predictions reduces the RMS down to 115 keV. The residuals are shown in Fig. 4. We are unaware of any unbiased RMS values that are this low, including from advanced AI models. (Note that some AI works include training-data nuclei in their RMS numbers, which can be highly biased.)

Table S1 shows how our procedure compares with using the GK relations directly to correct WS4. For nuclei that have nearly all nearest and next-to-nearest neighbors observed, the local Jaffe procedure is similar to GK using a mean of all 12 GK-type predictions for each nucleus [55]. The real advantage of our procedure over using the GK relations directly is that local Jaffe corrections also work well when most of the neighboring nuclei are not measured, which is the most interesting region. As noted in the Letter, scientifically what is desired is not higher-precision predictions of nuclei surrounded by well-measured neighbors, but trustworthy ways of extrapolating corrections away from the stable region. The local Jaffe approach provides an interpretable method for deriving such corrections.
